# LAP-like non-canonical autophagy and evolution of endocytic vacuoles in pancreatic acinar cells

**DOI:** 10.1080/15548627.2019.1679514

**Published:** 2019-10-25

**Authors:** Francesca De Faveri, Michael Chvanov, Svetlana Voronina, Danielle Moore, Liam Pollock, Lee Haynes, Muhammad Awais, Alison J. Beckett, Ulrike Mayer, Robert Sutton, David N. Criddle, Ian A. Prior, Tom Wileman, Alexei V. Tepikin

**Affiliations:** a Department of Cellular and Molecular Physiology, University of Liverpool, Liverpool, UK; b Department of Molecular and Clinical Cancer Medicine, University of Liverpool, Liverpool, UK; c Bio-Medical Research Centre, Norwich Medical School, Faculty of Medicine and Health Sciences, University of East Anglia, Norwich, UK

**Keywords:** Actin, acute pancreatitis, autophagy, endocytic vacuoles, endocytosis, exocytosis, LAP, LC3, non-canonical autophagy, pancreas, pancreatic acinar cells

## Abstract

Activation of trypsinogen (formation of trypsin) inside the pancreas is an early pathological event in the development of acute pancreatitis. In our previous studies we identified the activation of trypsinogen within endocytic vacuoles (EVs), cellular organelles that appear in pancreatic acinar cells treated with the inducers of acute pancreatitis. EVs are formed as a result of aberrant compound exocytosis and subsequent internalization of post-exocytic structures. These organelles can be up to 12 μm in diameter and can be actinated (i.e. coated with F-actin). Notably, EVs can undergo intracellular rupture and fusion with the plasma membrane, providing trypsin with access to cytoplasmic and extracellular targets. Unraveling the mechanisms involved in cellular processing of EVs is an interesting cell biological challenge with potential benefits for understanding acute pancreatitis. In this study we have investigated autophagy of EVs and discovered that it involves a non-canonical LC3-conjugation mechanism, reminiscent in its properties to LC3-associated phagocytosis (LAP); in both processes LC3 was recruited to single, outer organellar membranes. Trypsinogen activation peptide was observed in approximately 55% of LC3-coated EVs indicating the relevance of the described process to the early cellular events of acute pancreatitis. We also investigated relationships between actination and non-canonical autophagy of EVs and concluded that these processes represent sequential steps in the evolution of EVs. Our study expands the known roles of LAP and indicates that, in addition to its well-established functions in phagocytosis and macropinocytosis, LAP is also involved in the processing of post-exocytic organelles in exocrine secretory cells.

**Abbreviations:**

AP: acute pancreatitis; CCK: cholecystokinin; CLEM: correlative light and electron microscopy; DPI: diphenyleneiodonium; EV: endocytic vacuole; LAP: LC3-associate phagocytosis; MAP1LC3/LC3: microtubule-associated protein 1 light chain 3; PACs: pancreatic acinar cells; PFA: paraformaldehyde; PtdIns3K: phosphatidylinositol 3-kinase; PtdIns3P: phosphatidylinositol 3-phosphate; Res: resveratrol; TAP: trypsinogen activation peptide; TEM: transmission electron microscopy; TLC-S: taurolithocholic acid 3-sulfate; TRD: Dextran Texas Red 3000 MW Neutral; ZGs: zymogen granules.

## Introduction

Pancreatic acinar cells (PACs) secrete digestive enzymes and precursors of digestive enzymes (zymogens). In PACs these secretory proteins are packaged in large, optically dense zymogen granules (ZGs). The important secretagogs CCK (cholecystokinin) and acetylcholine utilize Ca^2+^ signaling cascades to trigger and regulate secretion in this cell type [1–4]. Secretion occurs as a result of compound exocytosis of ZGs involving both fusion of ZGs with the plasma membrane and intergranular fusion [5]. Post-exocytic Ω-shaped structures are produced as a consequence of such compound exocytosis [5–7]. The process of secretion involves rearrangement of cellular actin [6,8–11], which also interacts with post-exocytic structures [11]. The disconnection of post-exocytic structures from the plasma membrane results in formation of endocytic vacuoles (EVs) [7,12].

Under normal physiological conditions activation of zymogens occurs in the intestine, where digestive enzymes fulfill their physiological function. However, during acute pancreatitis (AP) activation of zymogens occurs in the pancreas itself, initiating autodigestion of the gland [13,14]. Pancreatic trypsinogen activation has been extensively documented and considered to be important for the pathophysiology of AP [13,15–20]. In our previous studies we observed trypsinogen activation in EVs [7,12]. Furthermore, we found that EVs can undergo rupture and fusion with the plasma membrane [12] providing a conduit for the release of digestive enzymes into the cytosol of the PACs and extracellular milieu. Characterization of the intracellular processing of EVs could therefore lead to a better understanding of early cellular events in the initiation of AP and pinpoint new molecular targets for disease treatment.

In the current study we aimed to characterize the relationships between EVs and macroautophagy/autophagy (which will be subsequently referred to as autophagy in this paper). Autophagy involves formation of membrane bound organelles known as autophagosomes, fusion of autophagosomes with lysosomes and degradation of cellular components contained in these hybrid organelles. Autophagy is vital for the normal homeostasis of the exocrine pancreas [21–23]. An increase in the number of autophagic vacuoles in PACs was clearly documented in animal models of AP and in isolated cells stimulated by the inducers of AP [24–26]. MAP1LC3/LC3 (microtubule-associated protein 1 light chain 3) has an important role in autophagy (reviewed in [27]). Conversion of LC3 from its cytosolic form LC3-I to its vesicular form LC3-II is utilized as a biochemical indicator of autophagosome abundance [28]. In *in vivo* models of AP the increase in the number of autophagic vacuoles in PACs was accompanied by increased levels of pancreatic LC3-II [24,25]. Interestingly, Hashimoto and colleagues described autophagic vacuoles containing ZGs [24]. This finding was later supported by the observed colocalization between LC3 and amylase [25]. The mechanism of ZG autophagy was further characterized by Vaccaro’s laboratory and the term “zymophagy” was coined to define this phenomenon [29]. The exact role of autophagy in the death/damage of PACs in conditions of AP is controversial, with evidence for both damaging [24] and protective [25,29,30] roles, although all studies emphasize the importance of this mechanism for AP.

Canonical autophagy involves engulfment of a cytoplasmic component by a phagophore, which leads to the formation of a double membraned autophagosome; this is followed by the fusion of the autophagosome with a lysosome (i.e. formation of autolysosome) and degradation of the luminal cargo (see [31] for a detailed explanation of the terminology). LC3 and its conjugation machinery, including ATG12–ATG5-ATG16L1 complex, is involved in canonical autophagy ([32,33], reviewed in [34]). The ATG16L1 recruitment step of canonical autophagy requires phosphatidylinositol 3-phosphate (PtdIns3P), produced by phosphatidylinositol 3-kinase (PtdIns3K) [35,36] and PtdIns3P effector WIPI2 [33].

In addition to canonical autophagy there is another LC3-dependent mechanism for producing a degradative membrane-bound compartment. This mechanism is termed LC3-associated phagocytosis (LAP) and it represents one of the forms of non-canonical autophagy ([37,38] for review see [39]). Importantly, under conditions of LAP, a phagophore is not required and LC3 is recruited directly to single-membrane organelles [37,38,40]. LC3 conjugation to the surface of phagosomes promotes lysosomal fusion and cargo degradation (reviewed in [39]). Notably, single-membrane LC3 lipidation also occurs on other endocytic compartments such as macropinosomes and entotic vacuoles [40–42]. These findings indicate that LAP-like non-canonical autophagy is a phenomenon involved in cargo degradation in the cytoplasmic vacuoles of diverse origins. Interestingly, canonical and non-canonical autophagy share many components of LC3 lipidation machinery including ATG5, ATG7, and ATG16L1 [37,40–42]. LAP, however, does not involve an ULK-ATG13-RB1CC1/FIP200 complex required for starvation-induced canonical autophagy [40]. LAP-like autophagy and canonical autophagy are also different in their responses to V-ATPase inhibitor bafilomycin A_1_, which facilitates LC3-II accumulation in canonical autophagy (by interrupting autophagic flux), while it prevents LC3-II formation in non-canonical autophagy [41,43]. A recent study by Fletcher and colleagues identified an important difference in the domains of ATG16L1 responsible for LC3 lipidation in canonical and LAP-like non-canonical (single membrane) autophagy. Of particular importance was the finding that a WD repeat-containing C-terminal domain (WD40 domain) is required for non-canonical autophagy but is dispensable for canonical autophagy [42]. Another difference identified in this study was the insensitivity of the single-membrane LC3 lipidation to the PtdIns3K inhibitor wortmannin, which efficiently suppressed canonical autophagy [42].

In this study we have identified autophagy of EVs and utilized distinctive properties of canonical and non-canonical autophagy to characterize and classify this process.

## Results

### Endocytic vacuoles form LC3-positive organelles

In our experiments EVs were identified by the fluorescence of membrane-impermeant probes (specified in individual figures) [12]; LC3 conjugation to EVs was revealed by fluorescence of GFP-LC3 expressed in all cells of transgenic GFP-LC3 mice (developed by Mizushima and colleagues [44]), including PACs (see Materials and Methods section for further details).

**Figure 1.**
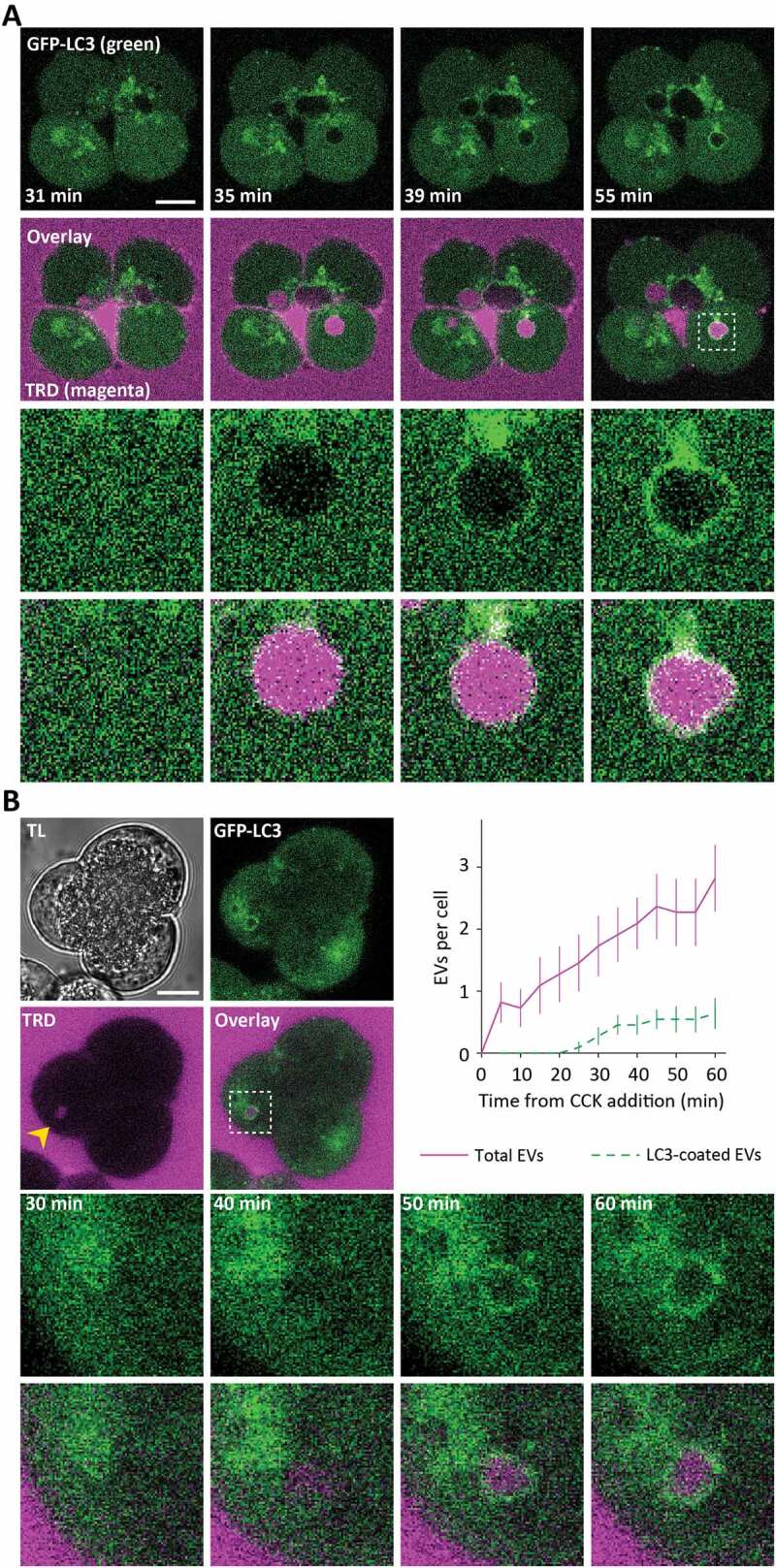
Endocytic vacuoles induced by CCK stimulation become coated by LC3. (**A**) The upper panels illustrate the formation and evolution of endocytic vacuoles (EVs) in pancreatic acinar cells (PACs) isolated from LC3-GFP transgenic mouse (GFP fluorescence is shown by green color) and stimulated by 500 pM of CCK in the presence of Texas Red labeled 3000 MW dextran (TRD); TRD fluorescence is shown by magenta color. Scale bar: 10 µm. The dashed box in the right panel of the second row of images highlights the fragment, shown on expanded scale in the 2 bottom rows of panels. The dynamics of formation and LC3 coating of the EVs in this group of cells is shown in the Movie S1A. (**B**) This part of the figure illustrates delay between the formation and LC3 coating of EVs. GFP-LC3 PACs were stimulated with 100 pM CCK in the continuing presence of TRD at 34.5°C and imaged every 5 min. TL indicates transmitted light image. Scale bar: 10 µm. A representative image of GFP-LC3 PACs (green), taken after 60 min of stimulation, is shown in the upper right panel. The second row of images shows TRD fluorescence (magenta); EV is indicated by the yellow arrowhead. Overlay of TRD and GFP-LC3 images (middle right panel) includes dashed box highlighting the fragment shown on the expanded scale in the bottom row of panels. Bottom panels show the fragment on the expanded scale at indicated times (illustrating formation and LC3 coating of the EV). Time graph displays the number of EVs per cell (mean ± SEM, magenta) and the number of LC3-EVs per cell (green) in PACs stimulated with 100 pM CCK; n_c_ = 11 cells, N = 5 mice.

This project was initiated by the discovery of LC3 conjugation to EVs formed in CCK-stimulated PACs (Figure 1A and Movie S1). Resolvable LC3 coating of EVs developed after approximately 20 min (Figure 1B) from the time of EV formation. It is important to note that CCK-stimulated cells can simultaneously contain both uncoated and LC3-coated EVs. The proportion of LC3-coated EVs (LC3-EVs) in the cells stimulated by 100 pM CCK for 30 min was approximately 7 – 20%. This value varied between experimental groups and will be specified for individual experimental protocols.

**Figure 2.**
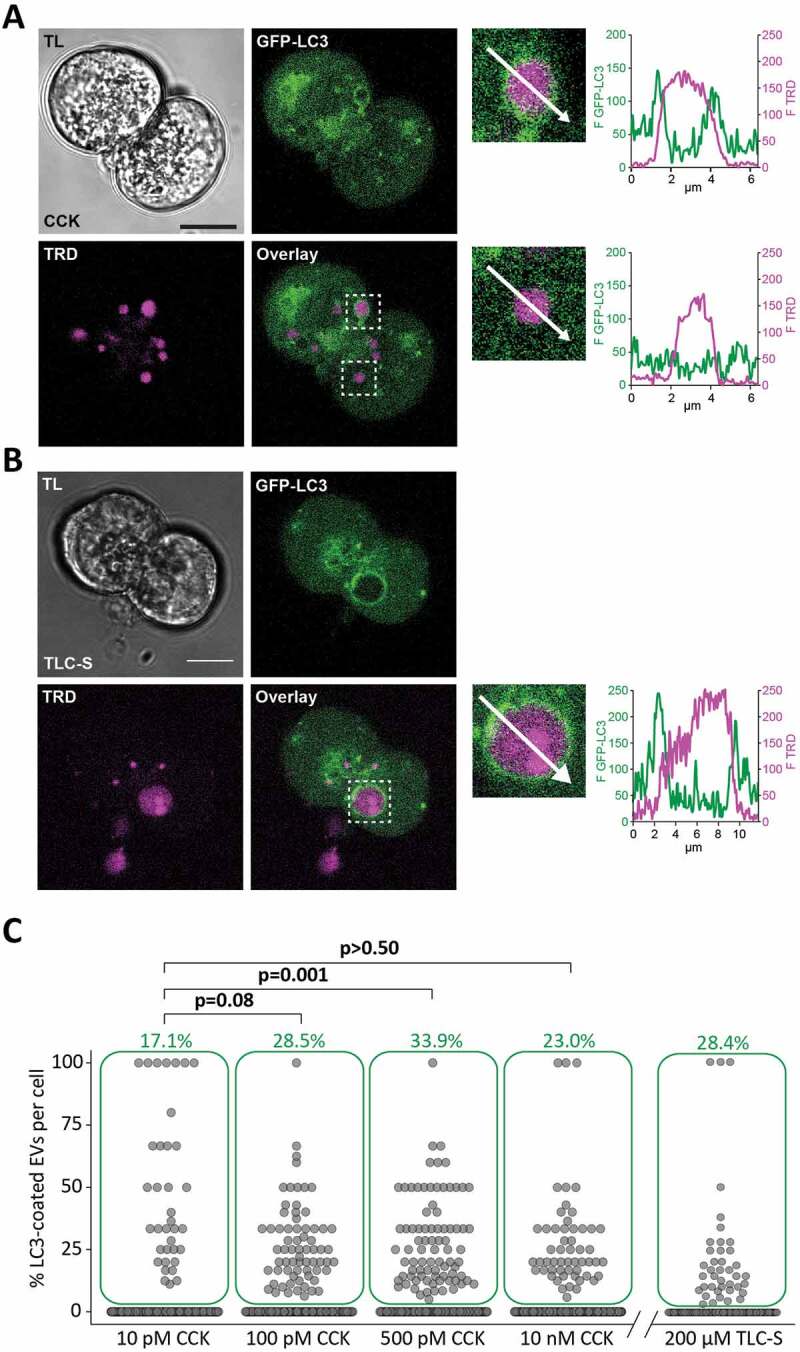
LC3 coats endocytic vacuoles induced by CCK and TLC-S. (**A**) GFP-LC3 PACs were stimulated with 500 pM CCK for 30 min at 34.5°C in the presence of TRD. TRD was removed from the extracellular solution before the beginning of imaging. TL indicates transmitted light image. Scale bar: 10 µm. A representative image of GFP-LC3 (green) PACs is shown in the upper right large panel. Lower left panel (labeled TRD) shows EVs in these cells (magenta). Overlay of TRD and GFP-LC3 images (lower large right panel) includes dashed boxes highlighting the fragments shown on the expanded scale on the small right panels (upper box corresponds to the upper fragment and illustrates LC3 coated EV; lower box corresponds to the lower fragment and illustrates EV not coated with LC3). Fluorescence intensity profiles plotted along the arrows are shown on the corresponding graphs. (**B**) GFP-LC3 (green) PACs were stimulated with 200 µM TLC-S for 20 min at 34.5°C in the presence of TRD. TRD was removed from the extracellular solution before the beginning of imaging. TL indicates transmitted light image. Scale bar: 10 µm. Representative image of GFP-LC3 (green) PACs is shown on the upper right large panel. Lower left panel (labeled TRD) shows EVs in these cells (magenta). Overlay of TRD and GFP-LC3 images (lower large right panel) includes dashed box highlighting the fragment shown on the expanded scale on the small right panel. The fragment illustrates LC3 coated EV. Fluorescence intensity profile plotted along the arrow is shown on the corresponding graphs. (**C**) The dot plot shows percentage of LC3-coated EVs formed in GFP-LC3 PACs stimulated with CCK in the presence of TRD. Each dot represents one cell and gives a percentage of LC3-coated EVs in this cell. The green boxes highlight cells with percentage of LC3-coated EVs above 0; the percentage of such cells is indicated above the boxes (green number). The rows of dots below the green boxes indicate cells which did not have LC3-coated EVs. In these experiments GFP-LC3 PACs were stimulated for 30 min at 35°C with 10 pM CCK (n_C_ = 211 cells; n_V_ = 728 EVs of which 55 LC3-coated [7.6%]), 100 pM CCK (n_C_ = 239 cells; n_V_ = 1381 EVs of which 105 LC3-coated [7.6%]), 500 pM CCK (n_C_ = 245 cells; n_V_ = 1330 EVs of which 119 LC3-coated [8.9%]) and 10 nM CCK (n_C_ = 235 cells; n_V_ = 1205 EVs of which n = 73 LC3-coated [6.1%]). N = 6 mice for all CCK concentrations. For the experiments with TLC-S, GFP-LC3 PACs were stimulated for 30 min at 35°C with 200 µM TLC-S (n_C_ = 141 cells; n_V_ = 1341 EVs of which 64 LC3-coated [4.8%]). N = 3 mice for TLC-S experiments.

The LC3-EVs were observed in cells stimulated with physiological (10 pM) and supramaximal (100 pM, 500 pM and 10 nM) concentrations of CCK for 30 min (Figure 2A and C). Note that the green numbers on the panel C indicate the proportion of cells with LC3-coated vacuoles and individual dots indicate the percentage of LC3-coated EVs per cell for individual cells. The proportion of LC3-EVs increased when the CCK concentration increased from 10 pM to 500 pM (Figure 2C). However, further increase in CCK concentration to 10 nM resulted in a modest but statistically significant decrease in the proportion of LC3-EVs. Total numbers of EVs for the specified CCK concentrations are shown in the Figure S1A. The proportion of LC3-EVs that occurred over longer incubation periods with 100 pM CCK is shown in the Figure S2A. The proportion at 150 min and 180 min was smaller than for 60 min.

Application of the bile acid TLC-S resulted in vacuolization of PACs [12,45] (Figure S1B). We observed formation of LC3-EVs in cells stimulated with TLC-S (Figure 2B,C) indicating that this phenomenon is not restricted to CCK stimulation.

### LC3 conjugation to endocytic vacuoles is blocked by V-ATPase inhibitors, protonophores and chloroquine

**Figure 3.**
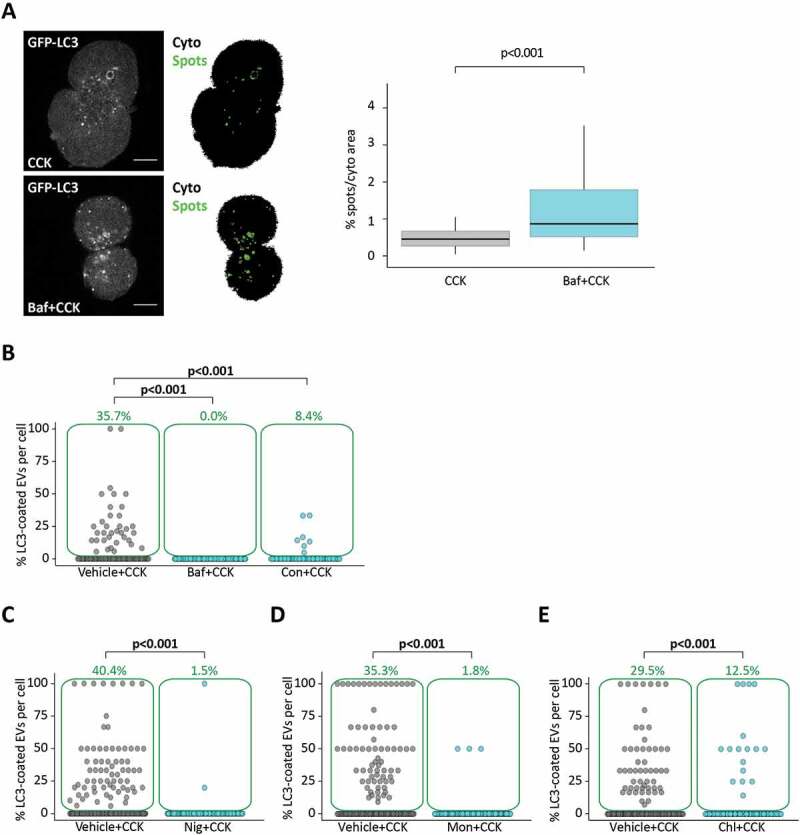
Disrupting acidification of intracellular organelles increases accumulation of LC3-GFP puncta but reduces LC3 coating of endocytic vacuoles. (**A**) GFP-LC3 PACs were incubated with 100 pM CCK for 30 min, after 30 min pre-incubation and in continuous presence of 0.1% DMSO (Vehicle + CCK, n_C_ = 83 cells) or 100 nM bafilomycin A_1_ dissolved in solution containing 0.1% DMSO (Baf+CCK, n_C_ = 76 cells); N = 4 mice for both conditions. Representative images of GFP-LC3 fluorescence (gray) and masks obtained as described in the Materials and Methods section (CytoArea = black, SpotsArea = green). The area of the cytoplasm occupied by GFP-LC3 hotspots is shown in the box plot. (**B**) Here and in parts C-D the dot plots show percentage of LC3-coated EVs formed in GFP-LC3 PACs stimulated with CCK in the presence of TRD. Each dot represents one cell and gives a percentage of LC3-coated EVs in this cell. The green boxes highlight cells with percentage of LC3-coated EVs above 0; the percentage of such cells is indicated above the boxes (green number). The rows of dots below the green boxes indicate cells which did not have LC3-coated EVs.

Experiments with the V-ATPase inhibitor bafilomycin A_1_ provided the first evidence that the observed LC3-EVs are formed by non-canonical autophagy. Bafilomycin A_1_ is frequently used as a tool to interrupt autophagic flux and study accumulating autophagosomes [28,46]. In our experiments, treatment with bafilomycin A_1_ increased the number of autophagosomes in CCK-stimulated PACs (Figure 3A). However, LC3 conjugation to EVs was completely eliminated by bafilomycin A_1_ (Figure 3B). Strong inhibition of LC3 conjugation to EVs was also observed in experiments with another inhibitor of V-ATPase concanamycin A (Figure 3B). Bafilomycin A_1_ induced a modest but statistically significant reduction in the total number of EVs, while concanamycin A had no effect on the total number of EVs (Figure S3A). Suppression of LC3 conjugation by bafilomycin A_1_ is one of the distinguishing properties of LAP [41,42]. In our experiments LC3 conjugation to EVs was also inhibited by the protonophores nigericin and monensin (Figure 3C,D correspondingly; Figure S4 shows a similar effect of prolonged incubation with monensin) and the acidotropic compound chloroquine (Figure 3E). Nigericin, monensin and chloroquine also reduced the total number of EVs in the PACs (Figure S3B-D correspondingly). The similarities of the effects of V-ATPase inhibitors, protonophores and chloroquine on LC3 conjugation to EVs suggest that acidic pH of EVs is important for the conjugation process.

### LC3 conjugation to endocytic vacuoles is insensitive to ULK1 inhibitors and phosphatidylinositol 3-kinase inhibitors

**Figure 4.**
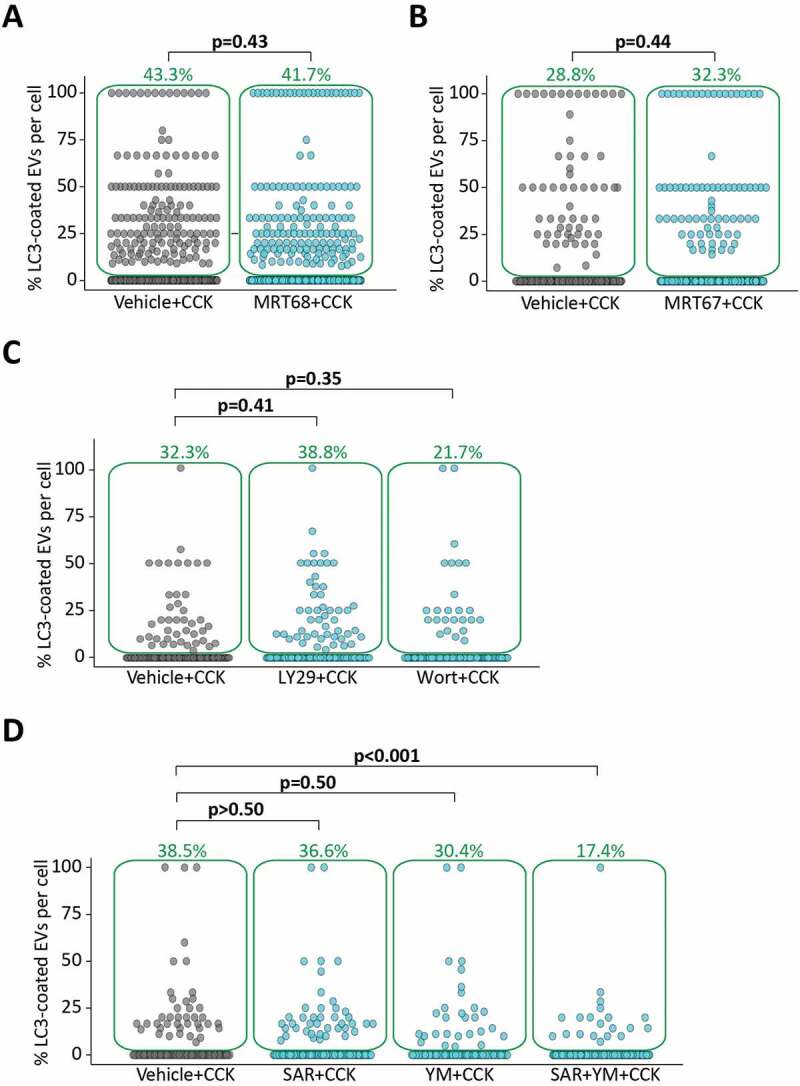
Inhibitors of canonical autophagy do not prevent LC3-coating of endocytic vacuoles. The dot plots in A-D show the percentage of LC3-coated EVs formed in GFP-LC3 PACs stimulated with CCK in the presence of TRD. Each dot represents one cell and gives a percentage of LC3-coated EVs in this cell. The green boxes highlight cells with percentage of LC3-coated EVs above 0; the percentage of such cells is indicated above the boxes (green number). The rows of dots below the green boxes indicate cells which did not have LC3-coated EVs. (**A**) GFP-LC3 PACs were stimulated with 100 pM CCK for 30 min, after 60 min pre-incubation and in continuous presence of 0.1% DMSO (Vehicle+CCK, n_C_ = 360 cells, n_V_ = 1747 EVs of which 253 LC3-coated [14.5%]) or 1 µM MRT68921 (MRT68+ CCK, n_C_ = 367 cells, n_V_ = 1688 EVs of which 210 LC3-coated [12.4%]). N = 5 mice for both conditions. MRT68921 had no resolvable effect on the total number of EVs in PACs (Fig. S6A). Note that MRT68921 had a strong and statistically significant effect on canonical rapamycin-induced autophagy in pancreatic acinar cells (see Fig. S5). (**B**) GFP-LC3 PACs were stimulated with 100 pM CCK for 30 min, after 60-min pre-incubation and in continuous presence of 0.1% DMSO (Vehicle+CCK, n_C_ = 205 cells, n_V_ = 639 EVs of which 103 LC3-coated [16.1%]) or 10 µM MRT67307 (MRT67+ CCK, n_C_ = 238 cells, n_V_ = 666 EVs of which 96 LC3-coated [14.4%]). N = 6 mice for both conditions. MRT67307 had no resolvable effect on the total number of EVs in PACs (Fig. S6B) (**C**) GFP-LC3 PACs were stimulated with 100 pM CCK for 30 min, after 30 min pre-incubation and in continuous presence of 1% DMSO (Vehicle+CCK, n_C_ = 130 cells, n_V_ = 899 EVs of which 60 LC3-coated [6.7%]) or 20 µM LY294002 (LY29+ CCK, n_C_ = 129 cells, n_V_ = 911 EVs of which 83 LC3-coated [9.1%]) or 20 µM wortmannin (Wort +CCK, n_C_ = 115 cells, n_V_ = 421 EVs, of which n = 31 LC3-coated [7.4%]). N = 3 mice for all conditions. The effects of the wortmannin and LY294002 on the total numbers of EVs are shown on the Fig. S7A. The effect of LY294002 on canonical rapamycin-induced autophagy was also tested (Fig. S5). Unlike LC3 conjugation to endocytic vacuoles, canonical autophagy was strongly inhibited by LY294002. (**D**) GFP-LC3 PACs were stimulated with 100 pM CCK for 30 min, after 30 min pre-incubation and in continuous presence of 0.1% DMSO (Vehicle+CCK, n_C_ = 104 cells, n_V_ = 629 EVs of which 51 LC3-coated [8.1%]) or 1 µM SAR405 (SAR+CCK, n_C_ = 112 cells, n_V_ = 723 EVs of which n = 52 LC3-coated [7.2%]) or 100 nM YM201636 (YM20+ CCK, n_C_ = 92 cells, n_V_ = 577 EVs of which 44 LC3-coated [7.6%]) or a combination of 1 µM SAR405 + 100 nM YM20 (SAR405+ YM20 + CCK, n_C_ = 115 cells, n_V_ = 733 EVs of which 26 LC3-coated [3.5%]). N = 3 mice for all conditions. SAR405 and YM201636 had no resolvable effect on the total number of EVs in PACs (Fig. S7B). .

Canonical, starvation-induced, autophagy requires ULK1 kinase [47,48] and is suppressed by ULK1 inhibitors (see [49] and Figure S5), while LAP is not dependent on ULK1 [40]. In our experiments LC3 conjugation to EVs was insensitive to the ULK1 inhibitors MRT68921 and MRT67307 (Figure 4 A,B, correspondingly). These results provide further support for the notion that the observed phenomenon represents non-canonical autophagy.

The phosphatidylinositol 3-kinase (PtdIns3K) PIK3C3/VPS34 is important for canonical autophagy (see [35,36] and Figure S5). The role of PtdIns3K in LAP is controversial (see [50] and [42]); presumably it depends on the specific LAP subtype. PtdIns3K is dispensable in some forms of LAP [42]. In our experiments PtdIns3K inhibitors LY294002 and wortmannin did not produce a resolvable reduction in the proportion of LC3-EVs (Figure 4C); SAR405 (recently developed selective inhibitor of PIK3C3 [51]) was also ineffective in reducing the proportion of LC3-EVs (Figure 4D). It was reported that phosphoinositide 5-kinase PIKFYVE can sustain autophagy in cells with inactivated PIK3C3 [52]. Therefore, we decided to test the effect of YM201636, an inhibitor of PIKFYVE. We found that YM201636 had no resolvable effect on the percentage of LC3-EVs when applied alone (Figure 4D). However, when YM201636 was applied in combination with SAR405 it induced a significant, although incomplete, decrease in the percentage of LC3- EVs (Figure 4D). These results suggest that depleting PtdIns5P in parallel to PtdIns3P suppresses this LC3 conjugation mechanism. In these experiments we found that SAR405 and YM201636 had no resolvable effect on the total number of EVs (Figure S7).

### LC3-conjugated endocytic vacuoles are single-membrane-bounded organelles

**Figure 5.**
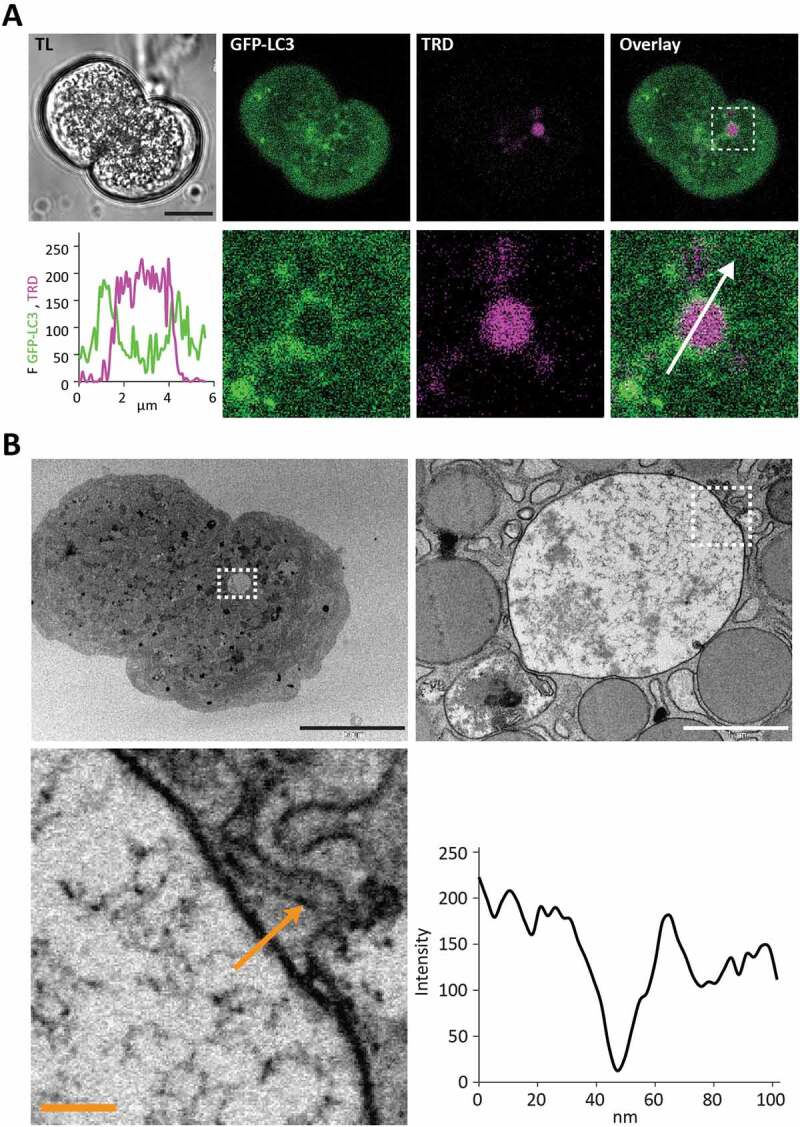
LC3-coated endocytic vacuoles have a single membrane. This figure shows correlation between live cell fluorescence images (A) and transmission electron microscopy (TEM) images (B) of the same cell containing a large LC3-coated endocytic vacuole. (**A**) Live cell images. TL indicates transmitted light image. Scale bar: 10 µm. GFP-LC3 (green) PACs were stimulated with 500 pM CCK in the presence of TRD (magenta). The endocytic vacuole (EV) selected for analysis is highlighted by a dashed box on the Overlay image (right panel in the top row). The region in the box is shown on the expanded scale in the low row of panels. The graph shows the intensity profile along the white arrow. (**B**) TEM images of the same cell and the same EV. Black scale bar corresponds to 10 µm, white scale bar corresponds to 1 µm, and yellow scale bar corresponds to 100 nm. The intensity profile along the yellow arrow is shown on the graph.

LAP involves LC3 conjugation to single membrane organelles; this is different from conventional macroautophagy, which requires formation and LC3 conjugation to double-membrane structures (reviewed in [39]). This structural difference could be useful in distinguishing the 2 phenomena. We therefore performed correlative light and electron microscopy (CLEM) of LC3-EVs. PACs expressing GFP-LC3 were placed on gridded coverslips and stimulated with 500 pM CCK in the presence of TRD (as described in Materials and Methods). We detected single membranes in all CLEM experiments with LC3-coated EVs (n = 8 cells and 8 EVs from N = 4 mice) (Figure 5). This finding supports the notion that LC3-EVs are formed by LAP-like non-canonical autophagy. Notably, in all of our samples, we were also able to clearly identify double-membraned organelles not correlated with EVs (Figure S8). The presence of these double membrane structures suggests that we are capable of resolving classical autophagy and that classical autophagosomes are structurally different from LC3-EVs.

### ROS generation is not essential for LC3 conjugation to endocytic vacuoles

**Figure 6.**
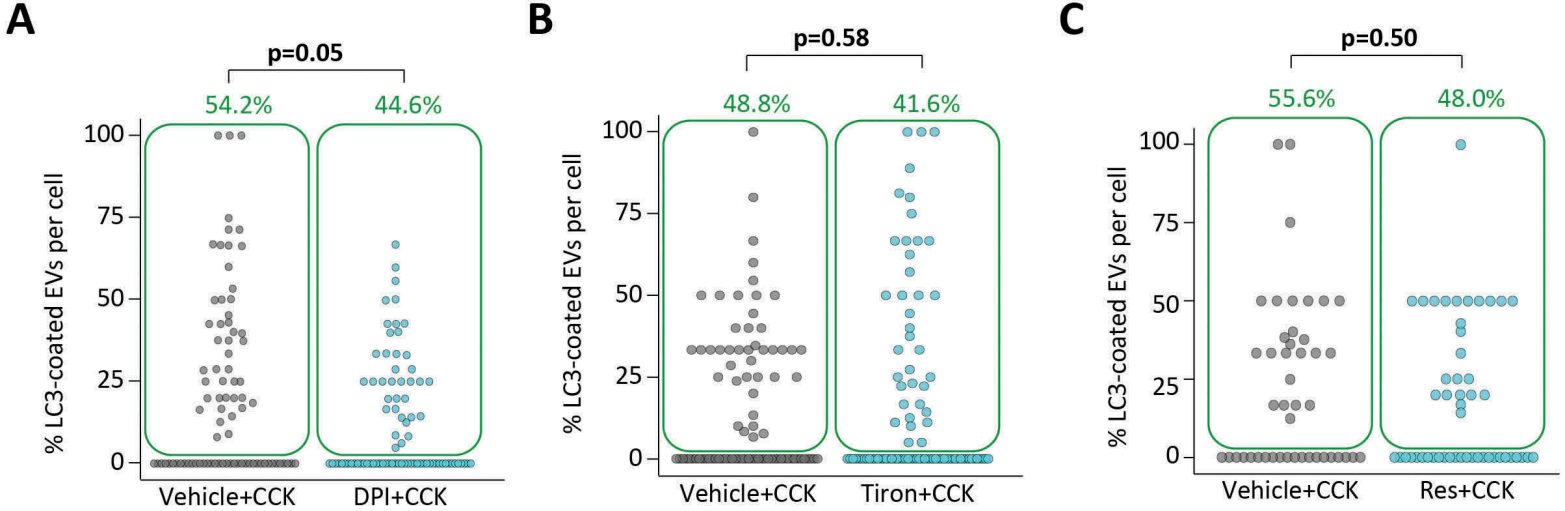
Effects of DPI, tiron and resveratrol on LC3 conjugation to endocytic vacuoles.

Generation of reactive oxygen species was shown to be important for some forms of LAP [50,53,54]; specifically, for LAP discovered and extensively characterized in macrophages [50]. Amongst experimental findings supporting this notion was the demonstration that the NADPH oxidase inhibitor diphenyleneiodonium (DPI) strongly suppressed LC3 conjugation to phagosomes [53–55]. In our experiments incubation of PACs with 10 μM of DPI resulted in only a modest reduction of LC3 conjugation to EVs in CCK-stimulated PACs; the effect was on the borderline of statistical significance (Figure 6A). In macrophages the antioxidant tiron completely inhibits LAP [50]. We have therefore tested a high concentration (1 mM) of tiron in our system and observed that the treatment with this antioxidant had no resolvable effect on LC3 conjugation to EVs (Figure 6B). Similar negative results were observed in experiments with the antioxidant resveratrol (Figure 6C), which was previously also shown to inhibit LAP [53]. The results of these experiments suggest that ROS generation is unlikely to be essential for LC3 conjugation to EVs. In these experiments we also found that DPI, tiron and resveratrol had no resolvable effect on the total number of EVs (Figure. S9).

### ATG16L1 is involved in LC3-EV formation: the role of the WD40 domain

**Figure 7.**
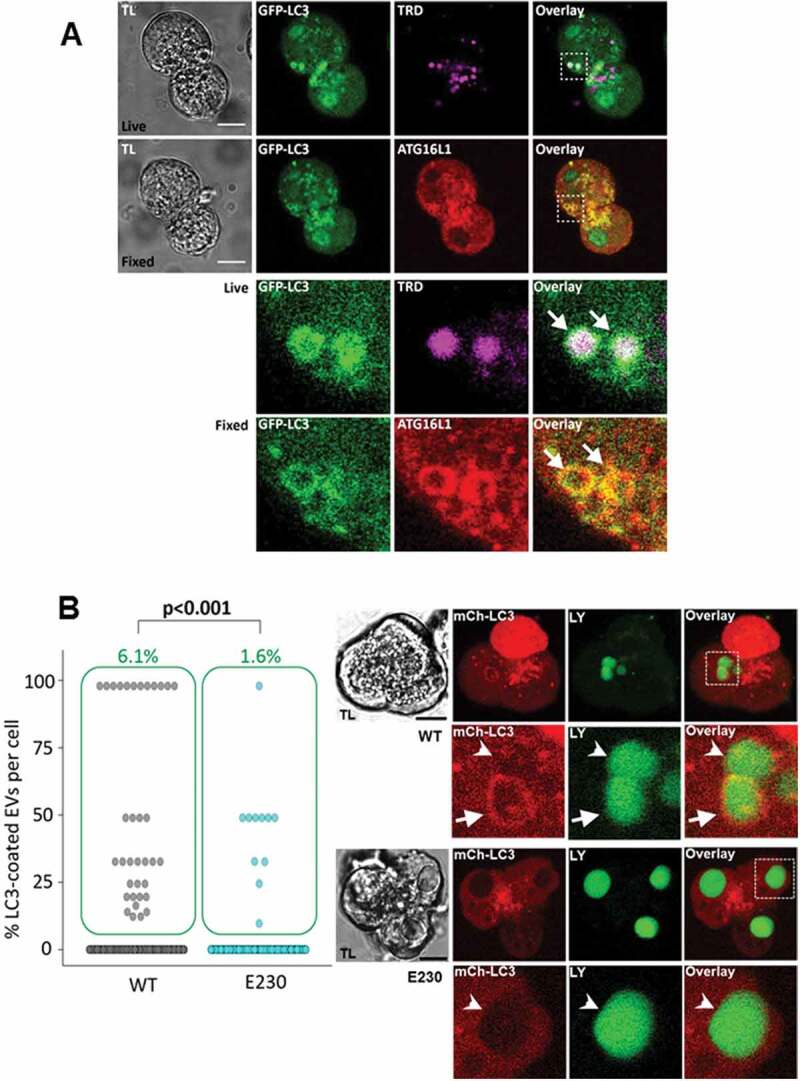
LC3-coating of endocytic vacuoles involves ATG16L1 and is suppressed in pancreatic acinar cells from Atg16L1^E230^ mice. (**A**) Correlative images of GFP-LC3 fluorescence in live cells and immunofluorescence labeling of ATG16L1 in fixed cells. TL indicates transmitted light images. Scale bars: 10 µm. In these experiments GFP-LC3 (green) PACs were stimulated with 500 pM CCK for 30 min in the presence of TRD (magenta to identify EVs) and imaged live on gridded dishes. They were then fixed with 4% PFA. Immunofluorescence staining for ATG16L1 (red) was performed as described in the Materials and Methods section. Correlative images of the same cells are shown: live cells (first [upper] row of images) and fixed cells (second row of images). Cellular region containing 2 LC3-coated EVs is highlighted by dashed boxes on the Overlay images of live and fixed cells; this region is shown on the expanded scale in the 2 bottom rows. White arrows point toward the LC3-coated EVs. Note colocalization of GFP-LC3 fluorescence with immunostaining for ATG16L1 on these organelles. In these experiments we analyzed 47 EVs and co-localization between GFP-LC3 and ATG16L1 immunostaining was observed in 45 of those EVs. Further description of correlative imaging of PACs before and after fixation is given in Supplementary material (see Fig. S10). (**B**) The dot plot shows the percentage of LC3-coated EVs formed in mCherry-LC3A expressing PACs (see images in the right panels), stimulated with 100 pM CCK for 30 min in the presence of Lucifer Yellow (LY). Each dot represents one cell and gives a percentage of LC3-coated EVs in this cell. The green boxes highlight cells with percentage of LC3-coated EVs above 0; the percentage of such cells is indicated above the boxes (green number). The rows of dots below the green boxes indicate cells which did not have LC3-coated EVs. The left part summarizes results of experiments with PACs from WT mice (N = 9 mice, n_C_ = 577 cells, n_V_ = 1416 EVs of which 35 were LC3-coated [2.5%]) and the right part illustrates outcome of experiments with PACs from Atg16L1^E230^ mice (abbreviated E230 on the figure; N = 11 mice, n_C_ = 668 cells, n_V_ = 1665 EVs of which 12 were LC3-coated [0.7%]). Images in the right panels illustrate EVs in PACs expressing mCherry-LC3A (mCh-LC3; red). Replication-deficient adenovirus was utilized to express mCh-LC3 in these PACs. The lower set of images was recorded from PACs derived from Atg16L1^E230^ mice (abbreviated as E230 in the figure); the upper set of images was recorded from PACs derived from WT mice. The cells were stimulated by 100 pM of CCK in the presence of LY; LY fluorescence is shown in green. TL indicates transmitted light image. Scale bars: 10 µm. The dashed boxes in the right (overlay) panels highlight the fragments, shown on the expanded scale below the complete images. The arrow on the WT fragment indicates an LC3-coated EV. Arrowheads on the WT and E230 fragments indicate EVs which were not coated by LC3. Only very few EVs in E230 cells were coated with mCherry-LC3 (0.7% of EVs; see the dot plot and accompanying text above); therefore, cells containing uncoated EVs (all 3 EVs in the E230 PACs shown in the figure are uncoated) were selected as representative for E230 cells. An example of an uncoated EV is also shown at an expanded scale (bottom row).

EVs are fragile and almost always rupture during fixation of PACs. We therefore developed the methodology of correlative pre- and post-fixation imaging (Figure S10), which allowed us to probe the localization of specific proteins on EVs. It is important to emphasize that such correlative imaging relies on GFP-LC3 fluorescence to identify the same organelle in live and fixed cells (Figure S10). Using this method, we observed the presence of ATG16L1 on LC3-coated EVs (Figure 7A). This finding suggests the involvement of the ATG12–ATG5-ATG16L1 complex in the LC3 conjugation to EVs. The presence of ATG16L1 on EVs does not, on its own, support canonical or non-canonical mechanism of LC3 conjugation to EVs, since ATG16L1 is involved in both mechanisms [42]. However, different domains of ATG16L1 are required for its canonical and non-canonical roles [42]. It has been recently shown that the WD40 domain is essential for the ATG16L1 role in non-canonical LC3 lipidation and transgenic mice (termed Atg16L1^E230^ mice) expressing WD40-defficient ATG16L1 have been developed [42,56]. The cells of these animals lack non-canonical autophagy (in particular, they are LAP defective), but are competent for canonical autophagy [42,56]. We isolated PACs from Atg16L1^E230^ mice and matched wild type (WT, littermates of Atg16L1^E230^) mice and utilized a replication deficient adenovirus to express mCherry-LC3 in these cells (images on Figure 7B). The cells were then stimulated with 100 pM CCK in the presence of LY. Viral transfection of PACs required short-term (up to 14 h) cell culture. The total number of EVs and the proportion of LC3-EVs in these experiments were smaller than in the freshly isolated PACs, probably reflecting a recognized deterioration of this cell type during short term culture/maintenance. Importantly, we did not find a significant difference in the total number of EVs (Figure S11) in the cells isolated from Atg16L1^E230^ and WT littermate mice but found a strong, statistically significant difference in the percentage of LC3-coated EVs (Figure 7B). The significantly smaller proportion of LC3-EVs in the cells isolated from Atg16L1^E230^ mice suggests that LC3 conjugation to EVs involves LAP-like non-canonical autophagy.

### Activation of trypsinogen in LC3-coated endocytic vacuoles

**Figure 8.**
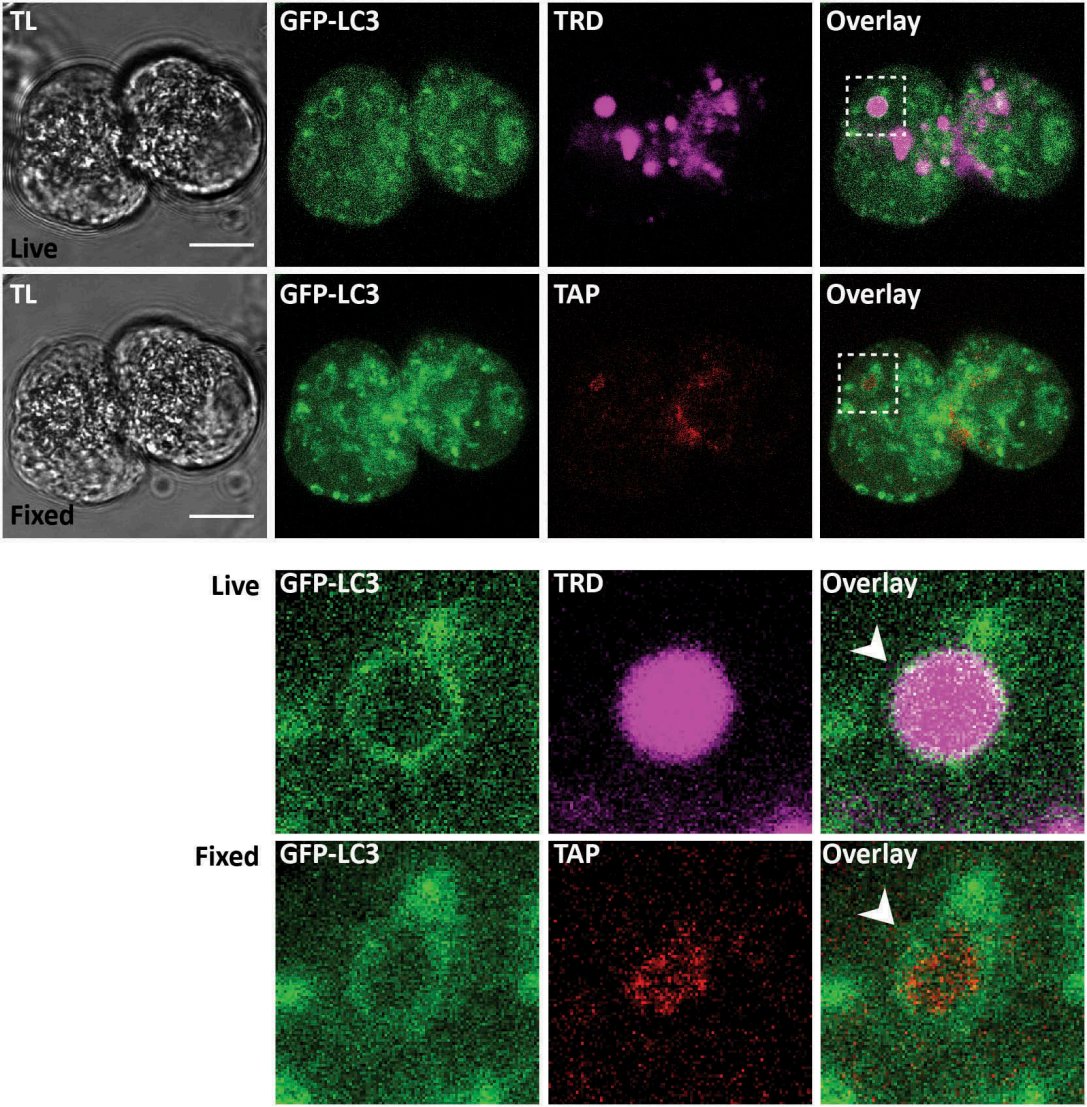
Trypsinogen activation peptide in LC3-coated endocytic vacuoles. The figure shows correlative images of GFP-LC3 fluorescence in live cells and immunofluorescence labeling of trypsinogen activation peptide (TAP) in fixed cells. TL indicates transmitted light images. Scale bars: 10 µm. In these experiments GFP-LC3 (green) PACs were stimulated with 500 pM CCK for 30 min in the presence of TRD (magenta to identify EVs) and imaged live on gridded dishes. They were then fixed with 4% PFA. Immunofluorescence staining for TAP (red) was performed as described in the Materials and Methods section. Correlative images of the same cells are shown: live cells (first (upper) row of images) and fixed cells (second row of images). Cellular region containing the LC3-coated EV is highlighted by dashed boxes on the Overlay images of live and fixed cells; this region is shown on the expanded scale in the 2 bottom rows. White arrowheads point toward the LC3-coated EV. Note co-localization of GFP-LC3 fluorescence with immunostaining for TAP in this organelle.

Correlative immunostaining was utilized to test the presence of trypsinogen activation peptide (TAP) in LC3-EVs. An example of such correlative staining is shown in Figure 8. We found TAP in a substantial proportion (26 out of 47) of successfully correlated LC3-EVs. The observed trypsinogen activation suggests retention (or at least partial retention) of digestive enzymes and precursors of digestive enzymes in LC3-EVs. This notion was further confirmed by correlative immunostaining for pancreatic amylase (see Figure S12), which was found in a substantial proportion (8 out of 10) of successfully correlated LC3-EVs.

### LC3 and F-actin do not co-exist on the same endocytic vacuoles

**Figure 9.**
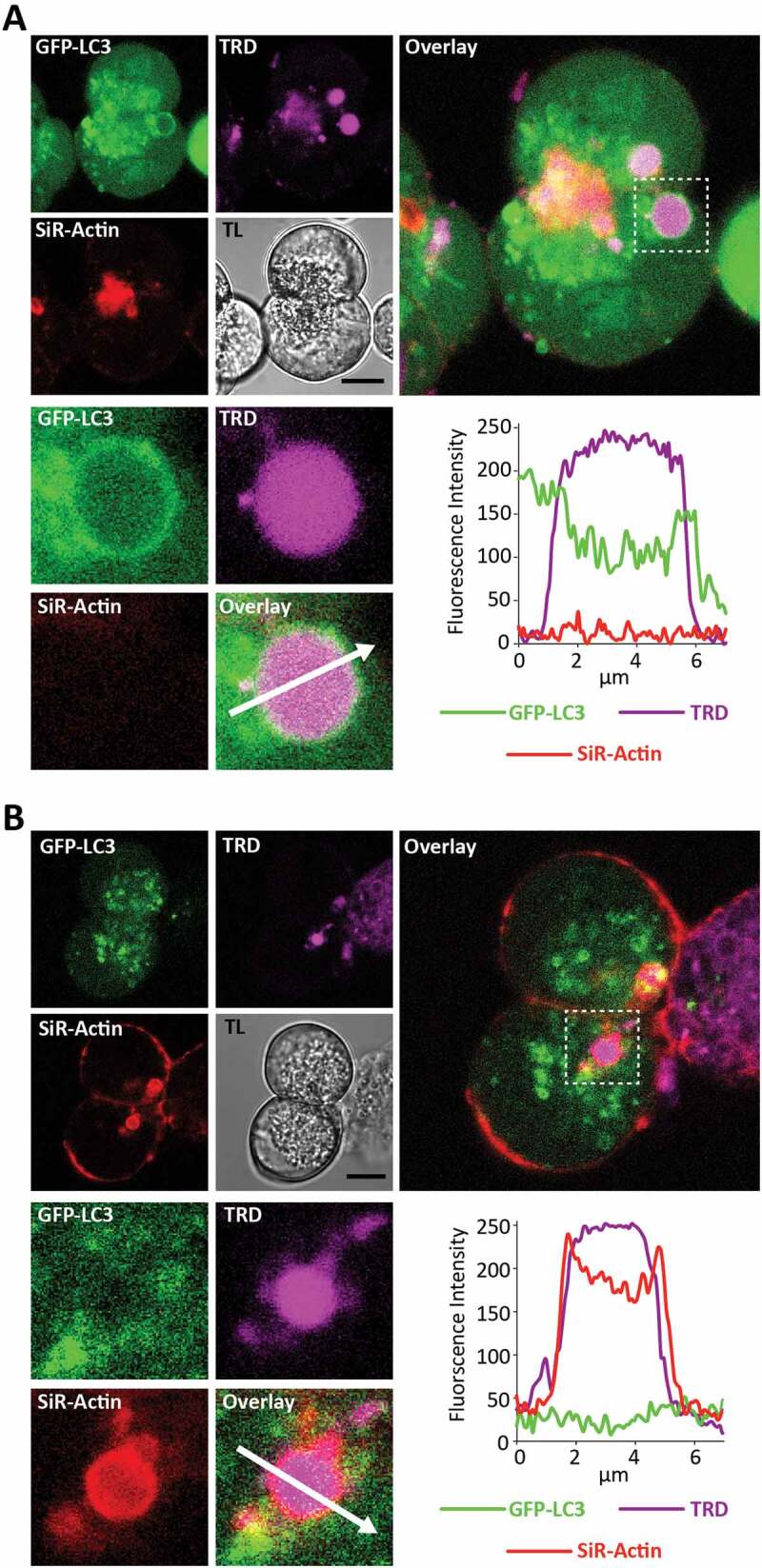
LC3-coated endocytic vacuoles are not coated by F-actin; F-actin-coated endocytic vacuoles are not coated by LC3. GFP-LC3 PACs were stained with SiR-Actin (to reveal F-actin distribution) and incubated for 30 min in the presence of 100 pM CCK and TRD. In these experiments we imaged 196 cells isolated from 8 mice. We analyzed 583 EVs, of which 47 were LC3-coated and 51 were actin-coated. There were no EVs labeled with SiR-Actin and GFP-LC3 simultaneously. (**A**) This part shows GFP-LC3 – positive EV, which was not stained with SiR-Actin. The image combines fluorescence of GFP-LC3 (green), TRD (magenta) and SiR-Actin (red). Scale bar is shown on the transmitted light (TL) image and corresponds to 10 µm. Selected EV with neighboring region of cytoplasm is highlighted by dashed square on the large right panel and shown on expanded scale in the lower panels. The fluorescence intensity profile along the arrow is plotted in the bottom right panel. EV is coated by LC3 but not by F-actin. (**B**) This part shows SiR-Actin -positive EV, which was not coated with GFP-LC3. The image combines fluorescence of GFP-LC3 (green), TRD (magenta) and SiR-Actin (red). Scale bar is shown on the transmitted light (TL) image and corresponds to 10 µm. Selected EV with neighboring region of cytoplasm is highlighted by dashed square on the large right panel and shown on expanded scale in the lower panels. The fluorescence intensity profile along the arrow is plotted in the bottom right panel. EV is coated by F-actin but not by LC3.

We previously reported that some EVs are coated by F-actin [12]. Considering the reported interaction of V-ATPases with F-actin [57] and the importance of V-ATPases for LAP-like autophagy ([41], see also Figure 3 in this paper), we decided to investigate whether LC3 and F-actin co-localize/interact on EVs. We therefore stained GFP-LC3 PACs with SiR-Actin and stimulated them with 100 pM CCK in presence of TRD. We counted total EVs, LC3-EVs and F-actin-coated EVs (F-actin-EVs). We analyzed 583 EVs (from 196 cells isolated from N = 8 mice), of which 47 were LC3-EVs and 51 were F-actin-EVs. We did not find any EVs coated by both LC3 and F-actin. Figure 9A shows an example of an LC3-EV, which has no F-actin staining. Figure 9B shows an example of an F-actin-EV, which does not have LC3. These experiments indicate that F-actin and LC3 do not simultaneously localize on EVs. We next measured the dynamics of F-actin and GFP-LC3 on EVs and observed that at the earliest time point (10 min) there were almost no LC3-EVs but F-actin-EVs were clearly resolved. The number of F-actin-EVs then decreased, accompanied by an increase in the number of LC3 – EVs (Figure S13). The results of our experiments suggest that F-actin-EVs and LC3-EVs represent different stages of EVs’ maturation.

## Discussion

In this study we visualized LC3 conjugation to EVs. Inhibition of this process by bafilomycin A_1_, single membrane conjugation identified by CLEM, insensitivity of the conjugation to PtdIns3K inhibitors and significantly reduced conjugation in the PACs from Atg16L1^E230^ mice suggest that the LC3 conjugation to EVs involves a non-canonical autophagy mechanism similar to LAP.

Our study revealed 3 stages of EV maturation in PACs: F-actin coated EVs (actinated EVs), uncoated EVs, and LC3-coated EVs (LC3-EVs) (see Figures 1, 2, 9 and Figure S13). F-actin plays an important role in stimulated secretion of zymogens from PACs [6,8–10]. Notably, post-exocytic structures, formed as a result of exocytosis of ZGs are coated with actin soon after their formation (i.e. after fusion of ZGs with the target membranes) [11]. This indicates that at the time of their formation EVs are actinated. Considering the delay between the formation and LC3-coating of EVs, as well as observed time-dependent changes in the numbers of actinated and LC3-coated EVs, we can conclude that the likely sequence of events is: actination of the EV (before and during its formation), loss of actin from the EV’s surface followed by LC3-conjugation to the EV membrane. The finding that the majority of EVs observed after 30 min of CCK stimulation were not coated with either F-actin or LC3 is consistent with this model.

It is interesting to note that the EVs’ ability to interact with F-actin at an early stage of their formation is shared with 2 other “classical” LAP-competent organelles – phagosomes ([37,40,50], actin interaction reviewed in [58]), and macropinosomes ([40,42], actin interaction reviewed in [59]). It is conceivable that in all 3 organelle types LAP developed from an evolutionally common mechanism established for dealing with potentially dangerous organellar cargo internalized by an actin-associated process. In addition to the reported similarities, also observed in this study, there are notable differences in the regulation of single membrane LC3 conjugation to the different organelles. An example of this is the difference in sensitivity of LC3 conjugation to ROS, which are required for LAP in macrophages [50,53] but are not essential for LC3 conjugation to EVs in PACs stimulated by CCK (Figure 6). This finding is consistent with previous observation that ROS generation in PACs stimulated by CCK is small and difficult to measure [60].

The presence of trypsin activity in intracellular organelles of PACs was documented biochemically [61,62] and later confirmed by fluorescence imaging (e.g. [17,26,63–65]). In our previous study we demonstrated that trypsinogen activation occurs in EVs [7,12]. This is consistent with the presence of TAP and amylase in EVs reported in this study. A probable explanation of this phenomenon is incomplete release of zymogens and digestive enzymes during compound exocytosis, leading to partial retention of these substances in EVs. We also cannot exclude the possibility that some zymogens and digestive enzymes are delivered into EVs as a result of fusion between these organelles with the secretory granules (e.g. which could occur soon after the closure of the fusion pore between the post-exocytic Ω-shaped structures and the plasma membrane). Finally, it is possible that in intact pancreas zymogens and digestive enzymes could be taken back up into EVs from the lumenal space (particularly in pathophysiological conditions involving congestion of pancreatic ducts and/or reduction of ductal secretion [66]). This could be relevant to conditions of acute and chronic pancreatitis. The latter mechanism, however, is unlikely to explain the presence of TAP and amylase in experiments on isolated cells (i.e. in conducted in this study) and the suggested partial retention of zymogens and digestive enzymes in EVs provides a reasonable explanation for the present findings. The presence of TAP in LC3-coated EVs, observed in this study (Figure 8), suggests interaction between LC3 conjugation and trypsinogen processing in these organelles. A recent study by Sender and colleagues indicated that macrophages phagocyte components of pancreatic acinar cells, including zymogen-containing vesicles, and that activation of trypsinogen occurs not only in PACs but also in macrophages [20]. Notably, LAP was originally identified as a mechanism involved in phagosome processing [37] and macrophages are prominently featured among LAP-competent cell types (e.g. [37] and [40]). It is therefore conceivable that LAP is the process responsible for trypsinogen activation in macrophages and that single membrane LC3 conjugation, in both PACs and macrophages, contributes to the pathophysiology of AP.

The transfer of luminally endocytosed material to autophagosomes in PACs was described in an influential paper by Lerch and colleagues [67]. We confirm this general observation and provide further important details indicating that the endocytic structure (i.e. EV) does not fuse with the autophagosome but is converted into a non-canonical autophagic organelle as a result of direct single-membrane LC3 conjugation.

In this study we observed strong inhibitory effects of bafilomycin A_1_ and concanamycin A on LC3 conjugation to EVs. These effects of V-ATPase inhibitors are consistent with the reported properties of other LAP-competent organelles [41] and support the notion that the observed LC3-conjugation to EVs occurs by a non-canonical mechanism. It should be further noted that V-ATPases are not static in PACs; indeed, Waterford and colleagues from Gorelick’s laboratory reported that caerulein (analogue of CCK used in this study) induces translocation of the V-ATPase V1 subunit from cytosolic to membrane fraction [68]. This translocation serves as a marker of V-ATPase activation. Furthermore, V-ATPase activity and organellar acidification was essential for caerulein-induced intracellular activation of zymogens [68,69]. Our findings and the results published by Gorelick’s laboratory [68] suggest an intriguing link between the translocation of V-ATPases, activation of zymogens and initiating of non-canonical autophagy. These relationships require further investigation and will be addressed in a separate study.

There was an interesting difference between the LC3 conjugation to EVs and to LAP-competent organelles in other cell types – in our experiments protonophores (nigericin and monensin) and weak base chloroquine strongly suppressed LC3 conjugation of EVs, while monensin and chloroquine potentiated non-canonical LC3-conjugation to endolysosomal compartments, phagosomes and entotic corpse vacuoles in cultured epithelial cells and in macrophages [40–42]. In these studies, “osmotic imbalance” was considered as the initiating stimulus for non-canonical LC3-conjugation. It is conceivable that in the EVs of PACs the nature of the osmotic imbalance is different. Zymogens are packaged and condensed in ZGs in an osmotically inert form [70]; putative incomplete release of zymogens during exocytosis [7,12] could result in conversion of retained zymogens from osmotically inert to osmotically active form inside EVs with the consequent increase of organellar osmolality. It is of course also possible that the signals for initiating LC3 conjugation to EVs of PACs and to LAP-competent organelles in other cell types are different.

A significant reduction in the proportion of LC3-EVs in PACs from LAP-deficient Atg16L1^E230^ mice provided additional evidence to support the notion that LC3 conjugation to EVs involves a non-canonical mechanism (see Figure 7). However, even in this definitive genetic model a small proportion of EVs was LC3 coated. This finding was surprising – it might have been expected that either no effect (if autophagy of EVs is canonical) or complete inhibition of LC3 conjugation (if autophagy of EVs is non-canonical) would be present. A possible explanation for the observed strong but incomplete inhibition is the redundancy of LC3-conjugating mechanisms involving fast non-canonical conjugation and slower canonical conjugation. In our experiments the number of LC3-coated vacuoles in PACs isolated from Atg16L1^E230^ mice was small and the mechanism of the LC3-conjugation to EVs in this model was difficult to investigate further.

The presence of ATG16L on EVs and high degree of co-localization between ATG16L1 and LC3 suggest that an ATG12–ATG5-ATG16L1 complex is involved in LC3 conjugation to EVs. This complex is also known to be involved in canonical autophagy (recently reviewed in [34]). The only difference identified between canonical and LAP-like non-canonical autophagy is defined by the domains involved in ATG16L1 binding to the organellar membranes [42]. A number of studies describe the importance of autophagy in the homeostasis of exocrine pancreas (e.g. [21]) and the pathophysiology of AP (e.g. [24–26]). Importantly, many experimental protocols used in these studies are expected to inhibit both canonical and LAP-like non-canonical autophagy (e.g. knockdown or knockout of *Atg5* [21,24,25]). Our study suggests that both canonical and non-canonical autophagy occur simultaneously in PACs. In the future it will be important to investigate the role of individual autophagy components in the physiology and pathophysiology of the exocrine pancreas.

## Materials and methods

### Materials

The following compounds were used: bafilomycin A_1_ (Bio-Techne, 1334); concanamycin A (Bio-Techne, 2656); monensin (Bio-Techne, 5223); nigericin (Bio-Techne, 4312); MRT67307 (Bio-Techne, 5134); MRT68921 (Stratech, 57949-SEL); LY294002 (Bio-Techne, 1130); wortmannin (Bio-Techne, 1232); collagenase (Sigma-Aldrich, C9407); poly-L-lysine (Sigma-Aldrich, P8920); sodium pyruvate (Sigma-Aldrich, S8636); Minimum Essential Medium (MEM; Sigma-Aldrich, M5550); penicillin-streptomycin-glutamine (ThermoFisher Scientific, 10378016); CCK (cholecystokinin) fragment 26–33 (Sigma-Aldrich, C2175); rapamycin (Sigma-Aldrich, 553210); YM201636 (Stratech, 51219-SEL); SAR405 (Cayman Chemical, 16976); paraformaldehyde (PFA; Agar Scientific, R1026); gluteraldehyde (Agar Scientific, R1020); OsO_4_ (Agar Scientific, R1024); potassium ferrocyanide (Sigma-Aldrich, P-3289); aspartic acid (Sigma-Aldrich A8949); thiocarbohydrazide (TAAB, T009); uranyl acetate (TAAB, U001); acetylated BSA (Aurion, 900.099); diphenyleneiodonium chloride (DPI; Sigma-Aldrich, D2926); resveratrol (Sigma-Aldrich, R5010); tiron (Sigma-Aldrich, D7389); SiR-actin (Spirochrome AG/tebu-bio, SC001; kit contains SiR-actin and verapamil); phalloidin-Alexa Fluor 568 (ThermoFisher Scientific, A12380); trypsin inhibitor from Glycine max (soybean; Sigma-Aldrich, T9003); digitonin (Sigma-Aldrich, D5628); goat serum (Sigma-Aldrich, G9023).

The following fluorescence probes were used for labeling EVs: Lucifer Yellow lithium salt (ThermoFisher Scientific, L453); Disulfo-Cy5 carboxylic acid (Cyandye LLC, 20010); Dextran, Texas Red™, 3000 MW, Neutral (ThermoFisher Scientific, D3329); Dextran, Texas Red™, 3000 MW, Lysine Fixable (ThermoFisher Scientific, D3328); Dextran, Texas Red™, 10,000 MW, Lysine Fixable (ThermoFisher Scientific, D1863) and Dextran, Alexa Fluor™ 647, 10,000 MW, Anionic, Fixable (ThermoFisher Scientific, D22914).

The following antibodies were utilized: anti-ATG16L1 rabbit polyclonal antibodies (MBL International Corporation, PM040); anti-Rabbit IgG–Alexa Fluor™ 647 (goat) (ThermoFisher Scientific, A21244); anti-pancreatic alpha amylase (Abcam, ab21156); anti-TAP antibody (Antibodies Online, ABIN1717122).

### Animals and procedures

GFP-LC3#53 mice (referred to as GFP-LC3 mice) were originally developed by N. Mizushima and colleagues [44] and were provided by the RIKEN BRC through the National Bio-Resource Project of the MEXT, Japan. RIKEN RRC reference number for these animals is RBRC00806. For the generation of GFP-LC3 mice founder animals (C57BL/6N Crj x BDF1) expressing GFP-LC3 were backcrossed to C57BL/6N Crj and maintained as heterozygotes for the gene of interest [44]. Animals were housed and bred in the Biomedical Services Unit at the University of Liverpool, and had *ad libitum* access to food and water.

Atg16L1^E230^ mice [42,56] and corresponding WT littermates were available from Professor Ulrike Mayer and Professor Thomas Wileman (University of East Anglia, UK). For the generation of Atg16L1^E230^ mice founder animals (C57BL/6N Crl x 129Sv) expressing Atg16L1^E230^ were backcrossed to C57BL/6J and maintained as heterozygotes for the genes of interest [42,56].

Animals were sacrificed by the Schedule 1 method of cervical dislocation, in accordance with the Animal (Scientific Procedures) Act 1986 (ASPA) and with approval by the University of Liverpool Animal Welfare Committee and Ethical Review Body (AWERB). Both female and male mice were used, at age 5–8 weeks.

### Primary pancreatic acinar cell isolation, culture and transfection

Pancreata were excised from sacrificed animals by dissection. Acinar cells were isolated by collagenase digestion (0.14–0.16 mg/mL). Isolated cells were seeded on poly-L-Lysine-coated glass-bottom 35 mm dishes (MatTek Corporation, Ashland, Massachusetts, USA) and kept in extracellular solution (140 mM NaCl, 4.7 mM KCl, 1.13 mM MgCl_2_, 10 mM 4-[2-hydroxyethyl]-1-piperazineethanesulfonic acid [HEPES; Sigma-Aldrich, H3375], 10 mM D-glucose, 1.2 mM CaCl_2_, pH 7.2–7.4). Specific compounds added to this extracellular solution were indicated.

For correlative experiments (examples shown in Figures 5, 7 and 8 see also Figure S10 and S12) we used poly-L-Lysine-coated glass-bottom 35 mm dishes (MatTek Corporation, Ashland, Massachusetts, USA) with gridded coverslips (see https://www.mattek.com/store/p35g-1-5-14-cgrd/) which allowed us to identify the selected cells and organelles after fixation.

For overnight culturing, cells were transferred into sterile-filtered culture medium (130 mM NaCl, 4.7 mM KCl, 1.13 mM MgCl_2_, 10 mM HEPES, 10 mM D-glucose, 1 mM CaCl_2_, 1 mM Na_3_PO_4_, 2 mM pyruvate, penicillin-streptomycin-glutamine 1X, Minimum Essential Medium [MEM] amino acids, 1 µM trypsin inhibitor, pH 7.4).

Adenoviral vector LC3A-mCherry (Vector Biolabs, Malvern, Pennsylvania, USA) was added at an indicated concentration 4 × 10^7^ PFU/ml and incubated at 35°C for 12–16 h.

### Labeling endocytic vacuoles

Only negligible numbers of EVs form in unstimulated PACs; after 30 min in TRD-containing solution only 27 EVs were observed in 124 unstimulated PACs (N = 4 mice), only one of those EVs was coated with GFP-LC3. The majority of unstimulated PACs (104 out of 124) had no EVs (see Figure S14 and S15). EVs are the consequence of stimulated compound exocytosis (e.g. [7]); in order to study EVs we therefore need to stimulate the cells with secretagogues. Exocytosis in PACs was stimulated by cholecystokinin fragment 26–33 (CCK) at 34.5–35.0°C. To reveal EVs cell-impermeable fluorescent indicators were added to the extracellular solution at the time of the addition of this potent Ca^2+^-releasing secretagogue. Lucifer Yellow and disulfo-Cy5 were used in the study. These probes have similar molecular masses (MM) of approximately 0.5 kDa. We also utilized fluorescently-labeled dextrans Dextran Texas Red 3000 MM Neutral, Dextran Texas Red 3000 MM Lysine Fixable and Dextran Alexa Fluor 647 10000 MM Anionic Fixable. In our previous study we determined that the same EVs are labeled by disulfo-Cy5, Lucifer Yellow and fluorescent dextrans with MM ≤ 10 kDa. Indicators used to reveal EVs are specified in the description of individual experiments.

### Staining the actin cytoskeleton

To label actin filaments, cells were incubated for 1 h at 34.5°C in the presence of 1 µM SiR-Actin and 10 µM verapamil. Cells were then washed with extracellular solution and utilized for experiments. SiR-Actin is a jasplakinolide analog that binds actin filaments, verapamil is an efflux pump inhibitor utilized to increase the signal-to-noise ratio.

Another technique of actin staining was developed for the studies of the time-dependency of F-actin association with the endocytic vacuoles. In such experiments, PACs isolated from one mouse were divided into 4 dishes, each stimulated with 100 pM CCK in the presence of 100 µM Dextran Alexa Fluor 647 10000 MW Anionic Fixable. The time of the stimulation varied from 10 min to 120 min to determine the kinetics of changes in actin and LC3 on the EVs. After the stimulation cells were fixed with low concentration (1.8%) of PFA at 4°C overnight. Cells were then permeabilized with 0.025% digitonin and stained with phalloidin-Alexa Fluor 568 according to manufacturer’s instructions (final concentration 6.6 µM in PBS [137 mM NaCl, 2.7 mM KCl, 10mM NaH_2_PO_4_, pH = 7.4] for 30 min). Importantly, fixation with low concentration of PFA allows retention of dextran in EVs. However, in our experience, this technique is suitable for staining with labeled phalloidin but not for immunostaining (correlative life-fixed experiments were used for immunostaining experiments; see the next part of the method section and Figure S10).

### Immunofluorescence staining

PACs were fixed in 4% PFA for 10 min at room temperature (RT). Cells were permeabilized in 0.2% Triton-X 100 for 5 min at RT. Blocking solution (PBS, 10% [v:v] goat serum, 1% [w:v] bovine serum albumin [BSA; Sigma-Aldrich, A3294]) was added for 1 h at RT. Cells were then incubated with primary antibody dissolved in primary antibody solution (PBS, 5% [v:v] goat serum, 0.1% [v:v] acetylated BSA) for 1 h at RT. Secondary antibody diluted in PBS was added for 30 min at RT.

### Confocal microscopy

Confocal microscopy was performed on the following setups: TCS SL and SP2 AOBS (Leica Microsystems, Wetzlar, Germany), LSM 510 and LSM 710 (Zeiss, Oberkochen, Germany). The following objectives were used: 63 × 1.4 numerical aperture (NA) oil-immersion, 63 × 1.4 NA oil-immersion, 63 × 1.2 NA water-immersion and 63 × 1.4 NA oil-immersion, respectively. Live cell imaging experiments on Leica TCS SL and Zeiss LSM 710 were performed at 34.5°C, as these systems are equipped with temperature control units. Z-stack images were acquired with a 1 µm distance between optical slices. The pinhole was 1.8 airy units. GFP-LC3 was excited with a 488-nm laser line and emission collected between 500 and 530 nm. Texas Red was excited with a 543 nm laser line and emission collected between 560 and 630 nm. Alexa Fluor™ 647 was excited with a 633-nm laser line and emission collected between 650 and 750 nm. Lucifer Yellow was excited with a 458-nm laser line and emission collected between 500 and 565 nm. Cy5 was excited with a 633-nm laser line and emission collected between 650 and 750 nm. LC3A-mCherry was excited with a 543-nm laser line and emission collected between 550 and 600 nm. SiR-actin was excited with a 633-nm laser line and emission collected between 650 and 750 nm.

### Transmission electron microscopy

PACs expressing GFP-LC3 were placed on gridded coverslips and stimulated with 500 pM CCK in the presence of TRD. TRD containing LC3-EVs were then identified and the locations of the cells and LC3-EVs on the grids recorded.

Cell fixation (2 1-min on/off cycles, 100W, 20Hg) and staining (3 20-s on/off cycles, 100 W, 20 Hg) was performed in a Pelco BioWave® Pro (Ted Pella Inc., Redding, California, USA). Samples were fixed in 2.5% glutaraldehyde in 0.1 M phosphate buffer with pH 7.4. Samples were forward processed through several staining steps. The reduced osmium staining (2% [v:v] OsO_4_, 1.5% [v:v] potassium ferrocyanide in ddH_2_O), facilitating fixation of lipids, was followed by mordant staining (1% [w:v] thiocarbohydrazide). At this point, a second osmium staining (2% [v:v] OsO_4_ in ddH_2_O) was performed. Samples were then stained with 1% (v:v) uranyl acetate over night at 4°C followed the next day by Walton’s Lead Aspartate (0.02 M lead nitrate, 0.03 M aspartic acid, pH 5.5). To prevent precipitation artifacts the samples were washed with ddH_2_O between each of the staining steps described. Samples were then dehydrated in ethanol gradient (30, 50, 70, 90, 100% [v:v]) on ice. Samples were then infiltrated in hard resin (TAAB, Reading, UK): 1:1 resin:EtOH for 30 min and 100% resin 2 times for 30 min. Samples were embedded in resin at 60°C for 16 h. For TEM, 70–74 nm serial sections were cut on a microtome (Leica Microsystems, Wetzlar, Germany) and collected onto Butvar (0.25% [v:v] in chloroform, TAAB, Reading, UK) plastic-covered Gilder 200 hexagonal copper grids (GG017/C, TAAB, Reading, UK). Selected cells and LC3-EVs were identified in the resin embedded samples with the help of grid coordinates and images acquired on a 120 kV Tecnai G2 Spirit BioTWIN (FEI, Hillsboro, Oregon, USA) using a MegaView III camera and analySIS software (Olympus, Germany).

### Image processing

Images were processed on the following softwares: Leica AF Lite, LSM Image Browser, Zen Lite, ImageJ (NIH). For presentation purposes, linear adjustments of brightness and contrast were performed in ImageJ. Full size images with appropriate scale bars are shown, cropped fragments are clearly stated. Quantitative analysis was carried out on raw, unprocessed images. EVs were manually counted. A macro was created for ImageJ to allow for semi-automated processing of GFP-LC3 fluorescence hotspots. The cytoplasm area was calculated by the pixels highlighted by a mask obtained by Huang method of thresholding [71]. Imaging was performed with the same zoom and averaging. The area occupied by GFP-LC3 fluorescence hotspots was determined by the pixels highlighted by a mask obtained by Maximum Entropy thresholding [72], followed by a particle restriction limit of 10-Infinity pixels. The percentage of the area occupied by hotspots divided by the cytoplasm area was calculated.

### Statistical analysis

In the Results section n_C_ represents the number of cells and N indicates the number of mice. Since we were interested in cellular events, individual cells were considered the prime subjects for statistical analysis. For some experiments we also included the number of analyzed EVs (n_V_).

Statistical analysis and graph generation were performed in R (http://www.r-project.org/). Firstly, data were tested for normality with Shapiro-Wilk test. Data not following a normal distribution are presented as box and whisker plots. The ends of the box represent the first and third quartiles; the thick line represents the median. The top whiskers represent the highest values equal or below the third quartile plus 1.5 times the inter-quartile range (IQR). Likewise, the bottom whiskers represent the lowest values equal or above the first quartile minus 1.5 times the IQR. When data distribution was extremely skewed, meaning that whiskers, percentiles and median all fell on the same value (this was frequently the case for analysis of cellular distributions of LC3-EVs), dot plots were presented instead. This format should allow a clear visualization of the raw data distribution and we feel that it represents the best way to present our type of data. Nonparametric statistics were used for analysis of data which did not show a normal distribution. The Wilcoxon-Mann-Whitney test (Wilcoxon rank sum test) was used to compare 2 groups of independent observations. The Kruskal-Wallis rank sum test was used to compare multiple groups of independent observations. Data was forward processed to a Dunn test for post-hoc analysis, to compare individual groups to control, with p-values adjusted with the Bonferroni method.

## Supplementary Material

Supplemental Material
