# Supplementary material for: LAP-like non-canonical autophagy and evolution of endocytic vacuoles in pancreatic acinar cells
Source: Autophagy. 2019 Oct 25;16(7):1314–31. doi: 10.1080/15548627.2019.1679514 (PMC7469629; doi:10.1080/15548627.2019.1679514)
Supplement: Supplemental Material [file KAUP_A_1679514_SM7417.zip › Supplementary information/Supplem LAP in Acinar Cells 29 Aug VFb.docx]

**Supplemental material**

**LAP-like non-canonical autophagy and evolution of endocytic vacuoles in pancreatic acinar cells**

**Movie S1.** LC3 conjugation to endocytic vacuoles. (**A**) The movie illustrates the dynamics of endocytic vacuoles (EVs) and LC3 conjugation to these organelles. The experiment was conducted on pancreatic acinar cells isolated from GFP-LC3 transgenic mouse (GFP fluorescence is shown by green color) and stimulated by 500 pM of CCK in the presence of Dextran Texas Red 3000 MW Neutral (TRD); TRD fluorescence in shown by magenta color. Note that TRD was first added to the extracellular solution (simultaneously with CCK) and later removed from the extracellular solution by perfusion. Intracellular TRD fluorescence reveals EVs. The movie supplements Figure 1 of the main manuscript. (**B**) The same movie as in (A) but without TRD fluorescence (i.e. magenta color removed) so that only GFP-LC3 fluorescence is visible (highlighting LC3 conjugation to intracellular organelles).

**Figure S1.** Total number of endocytic vacuoles in stimulated pancreatic acinar cells.

The figure supplements Figure 2 of the main manuscript. The box and whisker plots show total numbers of EVs per cell. PACs were stimulated with CCK or TLC-S in the presence of Dextran Texas Red 3000 MW Neutral (TRD). TRD was removed from the extracellular solution before the beginning of imaging. (**A**) CCK-induced formation of EVs. In these experiments GFP-LC3 PACs were stimulated for 30 min at 35°C with 10 pM CCK (n_C_=211 cells; n_V_=728 EVs), 100 pM CCK (n_C_=239 cells; n_V_ =1381 EVs), 500 pM CCK (n_C_=245 cells; n_V_ =1330 EVs) and 10 nM CCK (n_C_=235 cells; n_V_ =1205 EVs). N=6 mice for all CCK concentrations. (**B**) TLC-S-induced formation of EVs. In these experiments GFP-LC3 PACs were stimulated for 30 min at 35°C with 200 µM TLC-S (n_C_=141 cells; n_V_=1341 EVs). N=3 mice for TLC-S experiments.

**Figure S2.** Formation of endocytic vacuoles and LC3-conjugated endocytic vacuoles in a pancreatic acinar cell stimulated by CCK for longer periods of time (60-180 min). (**A**) Shows the percentage of LC3-coated EVs formed in GFP-LC3 PACs stimulated with CCK in the presence of TRD. Each dot represents one cell and denotes the percentage of LC3-coated EVs in this cell. The green boxes highlight cells with percentage of LC3-coated EVs above 0; the percentage of such cells is indicated above the boxes (green number). The rows of dots below the green boxes indicate cells which did not have LC3-coated EVs. In these experiments GFP-LC3 PACs were stimulated at 35°C with 100 pM CCK and imaged live after 60 min (n_C_=91 cells; n_V_=745 EVs of which 38 LC3-coated [5.1%]), 90 min (n_C_=81 cells; n_V_ =665 EVs of which 21 LC3-coated [3.2%]), 120 min 500 pM CCK (n_C_=86 cells; n_V_ =663 EVs of which 27 LC3-coated [4.1%]), 150 min (n_C_=84 cells; n_V_ =587 EVs of which n=9 LC3-coated [1.5%]) and 180 min (n_C_=90 cells; n_V_ =664 EVs of which n=11 LC3-coated [1.7%]). N=3 mice for all conditions. (**B**) The box and whisker plot show the total numbers of EVs per cell for this experiment.

**Figure S3.** Effect of V-ATPse inhibitors, protonophores and chloroquine on the total number of endocytic vacuoles in pancreatic acinar cells. The figure supplements Figure 3 of the main manuscript. The box and whisker plots show total numbers of EVs per cell. (**A**) Effects of V-ATPase inhibitors. Cells were stimulated with 100 pM CCK for 30 min, after 30 min pre-incubation and in continuous presence of 0.1% DMSO (Vehicle+CCK, n_C_ =98 cells, n_V_=571 EVs), 100 nM bafilomycin A_1_ (Baf+CCK, n_C_ =105 cells, n_V_ =451 EVs) or 100 nM concanamycin A (Con+CCK, n_C_ =83 cells, n_V_ = 415). (**B**) Effect of nigericin. Cells were stimulated with 100 pM CCK for 30 min, after 30 min pre-incubation and in continuous presence of 0.2% ethanol (Vehicle+CCK, n_C_=178 cells, n_V_ =875 EVs) or 1 µM nigericin (Nig+CCK, n_C_ =133 cells, n_V_ =388 EVs). N=3 mice for both conditions. (**C**) Effect of monensin. Cells were stimulated with 100 pM CCK for 30 min, after 60 min pre-incubation and in continuous presence of 0.1% ethanol (Vehicle+CCK, n_C_ =201 cells, n_V_=719 EVs) or 10 µM monensin (Mon+CCK, n_C_=170 cells, n_V_ =341). N=3 mice for both conditions. (**D**) Effect of chloroquine. Cells were stimulated with 100 pM CCK for 30 min, after 60 min pre-incubation and in continuous presence of 0.1% ethanol (Vehicle+CCK, n_C_=183 cells, n_V_=641 EVs) or 1 mM chloroquine (Chl+CCK, n_C_ =136 cells, n_V_=286 EVs). N=3 mice for both conditions.

**Figure S4.** Monensin inhibits LC3 conjugation to endocytic vacuoles. This figure supplements Figure 3 of the main manuscript and shows that the inhibitory effect of monensin on CCK-induced EVs is retained for prolonged periods of incubation with this protonophore. The dot plots in A-B show the percentage of LC3-coated EVs formed in GFP-LC3 PACs stimulated with CCK in the presence of TRD. Each dot represents one cell and denotes the percentage of LC3-coated EVs in this cell. The green boxes highlight cells with percentage of LC3-coated EVs above 0; the percentage of such cells is indicated above the boxes (green number). The rows of dots below the green boxes indicate cells which did not have LC3-coated EVs. (**A**) GFP-LC3 PACs were stimulated with 100 pM CCK for 60 min, after 30 min pre-incubation and in the continuous presence of 0.1% DMSO (Vehicle+CCK, n_C_ =81 cells, n_V_=311 EVs of which 42 LC3-coated [13.5%]) or 10 µM monensin (Mon+CCK , n_C_ =58 cells, n_V_ =222 EVs of which 2 LC3-coated [0.9%]). N=3 mice for both conditions. (**B**) GFP-LC3 PACs were stimulated with 100 pM CCK for 90 min, after 30 min pre-incubation and in the continuous presence of 0.1% DMSO (Vehicle+CCK, n_C_ =41 cells, n_V_=160 EVs of which 22 LC3-coated [13.8%]) or 10 µM monensin (Mon+CCK , n_C_ =74 cells, n_V_ =228 EVs of which 1 LC3-coated [0.4%]). N=3 mice for both conditions. The box and whisker plots in C-D show the total numbers of EVs per cell for the experiments described in A and B, respectively.

**Figure S5**. Effects of MRT68921 and LY294002 on rapamycin-induced autophagy in pancreatic acinar cells. This figure supplements Figure 4 of the main manuscript and indicates that, unlike LC3 conjugation to endocytic vacuoles, canonical autophagy in pancreatic acinar cells is suppressed by these compounds. The box plot shows the area of the cytoplasm occupied by GFP-LC3 hotspots. GFP-LC3 PACs were incubated with extracellular solution (Vehicle, n_C_ = 58 cells), 20 µM LY294002 (LY29, n_C_ = 46 cells) or 1 µM MRT68921 (MRT68, n_C_ = 54 cells) for 300 min, after 30 min pre-incubation and in the continuous presence of 0.1% DMSO or 100 nM bafilomycin A_1_ and 1 µM rapamycin (Rap+Baf, n_C_ = 80 cells, Rap+Baf+LY29, n_C_ = 29 cells, Rap+Baf+MRT68, n_C_ = 53 cells). N=3 mice for all conditions.

**Figure S6**. ULK1 inhibitors had no resolvable effect on formation of endocytic vacuoles in pancreatic acinar cells. The figure supplements Figure 4A and B of the main manuscript. The box and whisker plots show total numbers of EVs per cell. (**A**) MRT68921had no resolvable effect on EV formation. Cells were stimulated with 100 pM CCK for 30 min, after 60 min pre-incubation and in continuous presence of 0.1% DMSO (Vehicle+CCK, n_C_ =360 cells, n_V_=1747 EVs) or 1 µM MRT68921 (MRT68+CCK, n_C_ =367 cells, n_V_ =1688). N=5 mice for both conditions. (**B**) MRT67307 had no resolvable effect on EV formation. Cells were stimulated with 100 pM CCK for 30 min, after 60 min pre-incubation and in continuous presence of 0.1% DMSO (Vehicle+CCK, n_C_ =205 cells, n_V_ =639 EVs) or 10 µM MRT67307 (MRT67+CCK, n_C_ =238 cells, n_V_ =666 EVs). N=6 mice for both conditions.

**Figure S7.** Effects of phosphoinositide kinase inhibitors on formation of endocytic vacuoles in pancreatic acinar cells. The figure supplements Figure 4C and D of the main manuscript. The box and whisker plots show total numbers of EVs per cell. (**A**) Effects of PI3K inhibitors. Cells were stimulated with 100 pM CCK for 30 min, after 30 min pre-incubation and in continuous presence of 1% DMSO (Vehicle+CCK, n_C_ =130 cells, n_V_ =899 EVs) or 20 µM LY294002 (LY29+CCK, n_C_ =129 cells, n_V_ =911 EVs) or 20 µM wortmannin (Wort +CCK, n_C_ =115 cells, n_V_ =421 EVs). N=3 mice for all conditions. LY294002 had no resolvable effect on the EV numbers, whilst wortmannin reduced the number of EVs per cell. (**B**) Selective VPS34 and PIKFYVE inhibitors had no resolvable effect on EVs’ formation. Cells were stimulated with 100 pM CCK for 30 min, after 30 min pre-incubation and in continuous presence of 0.1% DMSO (Vehicle+CCK, n_C_ =104 cells, n_V_ =629 EVs) or 1 µM SAR405 (SAR+CCK, n_C_ =112 cells, n_V_ =723 EVs) or 100 nM YM201636 (YM20+CCK, n_C_ =92 cells, n_V_ =577 EVs) or a combination of 1 µM SAR405 + 100 nM YM20 (SAR405+YM20 + CCK, n_C_ =115 cells, n_V_ =733 EVs). N=3 mice for all conditions.

**Figure S8.** Example of an organelle with a double membrane. The figure supplements Figure 5 of the main manuscript. Representative transmission electron microscopy (TEM) images showing a double-membrane organelle. The organelle was selected from a TEM image of a pancreatic acinar cell stimulated with 500 pM of CCK for 30 min at 35°C. The organelle was not correlated with an endocytic vacuole or any other organelle from a live cell image. Black scale bar = 10 µm, white scale bar = 1 µm, yellow scale bar = 100 nm. The graph shows the intensity profile along the arrow. The figure illustrates our ability to resolve double-membrane structures of cellular organelles.

**Figure S9.** Diphenyleneiodonium, tiron and resveratrol have no effects on the formation of endocytic vacuoles. The box and whisker plots show the total numbers of EVs per cell. The figure supplements Figure 6 of the main manuscript. (**A**) Cells were stimulated with 100 pM CCK for 30 min, after 30 min pre-incubation and in continuous presence of 0.1% DMSO (Vehicle+CCK, n_C_ =83 cells, n_V_=417 EVs) or 10 µM DPI (DPI+CCK, n_C_ =83 cells, n_V_ =487 EVs). N=4 mice for both conditions. (**B**) Cells were stimulated with 100 pM CCK for 60 min, after 60 min pre-incubation and in the continuous presence of extracellular solution (described in Materials and Methods section) (Vehicle+CCK, n_C_ =86 cells, n_V_=160 EVs) or extracellular solution supplemented with 1 mM tiron (Tiron+CCK, n_C_ =89 cells, n_V_ =228). N=3 mice for both conditions. (**C**) Cells were stimulated with 100 pM CCK for 60 min, after 60 min pre-incubation and in the continuous presence of 0.1% DMSO (Vehicle+CCK, n_C_ =45 cells, n_V_=178 EVs) or 50 µM resveratrol (Res+CCK , n_C_ =50 cells, n_V_ =179 EVs). N=3 mice for both conditions.

**Figure S10.** Correlative images of LC3-coated endocytic vacuoles before and after fixation.

The figure supplements Figure 7A of the main manuscript. Endocytic vacuoles (EVs) are fragile and can undergo rupture even in unperturbed live cell [1]. Fixation with 4% of paraformaldehyde almost always ruptures the EVs and our attempts to reveal EVs in fixed cells using endocytosed fixable fluorescent-labelled (Dextran Texas Red 3000 MW Lysine Fixable or Dextran Alexa Fluor 647 10000 MW Anionic Fixable) were unsuccessful. This initially created problems with identification of EVs following immunofluorescence labelling. We resolved this problem by introducing the methodology of correlative pre- and post-fixation imaging illustrated in this figure. GFP-LC3 PACs were incubated for 30 min in the presence of 500 pM CCK and Dextran Texas Red 3000 MW Neutral (TRD), and the images of live cells were recorded. PACs were then fixed in 4% paraformaldehyde for 10 min at room temperature (RT), and washed three times with PBS. Images of fixed cells were recorded with the same confocal settings as utilized for the live cells. Scale bars are shown on the transmitted light (TL) images and correspond to 10 µm. *The upper row of cell images* shows EVs (identified by accumulated TRD) in live PACs. Some of these EVs are GFP-LC3 positive and others are negative. The dashed box on the overlay image indicates a large EV and the neighboring region of cytoplasm shown on the expanded scale (Live fragment). *The lower row of cell images* illustrates the loss TRD from EVs into the cytosol of the cells. Note the similarity in the distribution of GFP-LC3 in live and fixed cells, but drastic redistribution of TRD. The dashed box on the overlay image indicates a large EV (the same EV as in the upper row of images) and the neighboring region of cytoplasm shown on the expanded scale (Fixed fragment). Note that cellular debris visible in the right part of the live cell TL image were washed away during the fixation process (see TL image of fixed cells). *The upper row of vacuole images* (Live) shows a GFP-LC3 coated EV in a live cell. *The lower row of vacuole images* (Fixed) shows the same GFP-LC3 coated EV after fixation and loss of TRD. It is important to note that this correlative live-fixed cell imaging procedure is guided by GFP-LC3 fluorescence, which allows identification / correlation of GFP-LC3 conjugated EVs before and after fixation. This correlative live-fixed imaging of EVs which are not associated with GFP-LC3 is technically very challenging.

**Figure S11.** The total number of endocytic vacuoles is not different in pancreatic acinar cells from *ATG16L1^E20^* mice deficient for non-canonical autophagy (abbreviated E230 on the figure) and wild type (WT) mice. The box and whisker plot show total number of EVs per cell. The figure supplements Figure 7B of the main manuscript. In these experiments the pancreatic acinar cells (PACs) were incubated for 12-16 h with an adenoviral vector (see Materials and Methods section of the main manuscript for further details). The cells were then washed and stimulated with 100pm CCK for 30 min in the presence of Lucifer Yellow (LY) to visualize the endocytic vacuoles (EVs). The left part summarizes results of experiments with PACs from WT mice (N=9 mice, n_C_ = 577 cells, n_V_ = 1416 EVs) and the right part illustrates outcome of experiments with PACs from *ATG16L1^E20^* mice (abbreviated E230; N=11 mice, n_C_ = 668 cells, n_V_ = 1665 EVs). In these experiments the numbers of EVs per cell were smaller than in the freshly isolated PACs but similar in *ATG16L1^E20^* mice and WT mice, indicating that suppression of non-canonical autophagy does not affect EV formation.

**Figure S12.** Amylase in LC3-coated endocytic vacuoles. The figure shows correlative images of GFP-LC3 fluorescence in live cells and immunofluorescence labelling of amylase in fixed cells. TL indicates transmitted light images. Scale bars correspond to 10 µm. The figure supplements Figure 8 of the main manuscript. In these experiments GFP-LC3 (green) PACs were stimulated with 100 pM CCK for 30 min in the presence of TRD (magenta to identify EVs) and imaged live on gridded dishes. They were then fixed with 4% PFA. Immunofluorescence staining for amylase (red) was performed as described in the Materials and Methods section of the main manuscript. Correlative images of the same cells are shown: live cells (first [upper] row of images) and fixed cells (second row of images). The cellular region containing the LC3-coated EV is highlighted by dashed boxes on the Overlay images of live and fixed cells; this region is shown on the expanded scale in the two bottom rows. The white arrow points towards the LC3-coated EV. Note co-localization of GFP-LC3 fluorescence with immunostaining for amylase in this organelle.

**Figure S13**. Actin and LC3 on endocytic vacuoles. This figure supplements Figure 9 of the main manuscript and shows changes in the numbers of actinated and LC3-coated EVs. In these experiments GFP-LC3 PACs were stimulated with 100 pm of CCK in the presence of Alexa 647-dextran (Dextran, Alexa Fluor™ 647, 10,000 MW, Anionic, Fixable) to reveal the EVs. The cells were then fixed at the indicated time using a low concentration of PFA (1.8%, see Materials and Methods sections for further details). The presence of actin on endocytic vacuoles was revealed by staining with phalloidin-Alexa 568. The graphs show the mean numbers of actin-coated (red) and GFP-LC3 coated (green) EVs with corresponding SEM. Asterisks indicate statistical significance (p < 0.05, Dunnett’s test) of the difference between individual measurements and the corresponding first time point (10 min after the CCK addition). The results are based on the analysis of n_C_ = 456 cells. N= 5 mice for all conditions.

**Figure S14**. An example of unstimulated pancreatic acinar cells without endocytic vacuoles. The figure shows pancreatic acinar cells (PACs) isolated from GFP-LC3 transgenic mouse (GFP fluorescence is shown by green color) incubated in the presence of Texas Red-labelled 3000 MW dextran (TRD, magenta) for 30 min at 34.5°C and imaged without washing TRD. In this experiment cells were not stimulated with CCK and did not form endocytic vacuoles. Scale bar: 10 μm.

**Figure S15.** An example of unstimulated pancreatic acinar cells without endocytic vacuoles (following infection with replication-deficient adenovirus and prolonged incubation). In experiment shown on this figure PACs were isolated from the GFP-LC3 mouse. The cells were then infected with replication deficient adenovirus to express mCherry-tagged LC3 and incubated for 14 h in the extracellular solution described in the Material and Methods section of the main manuscript. The cells expressing GFP-LC3 (GFP fluorescence is shown by green color) and mCherry-LC3 (mCherry fluorescence is shown by red color) were incubated for 30 min in Cy5 - containing solution for 30 min at 34.5°C and then imaged without washing Cy5. In this experiment cells were not stimulated with CCK and did not form endocytic vacuoles (the figure is representative of 12 similar experiments). Scale bar: 10 μm.

**Refererences**

1. Chvanov M, De Faveri F, Moore D, et al. Intracellular rupture, exocytosis and actin interaction of endocytic vacuoles in pancreatic acinar cells: initiating events in acute pancreatitis. J Physiol. 2018 Jul;596(13):2547-2564. doi: 10.1113/JP275879. PubMed PMID: 29717784; PubMed Central PMCID: PMCPMC6023832.
