# Supplementary figures and images for: LAP-like non-canonical autophagy and evolution of endocytic vacuoles in pancreatic acinar cells
Source: Autophagy. 2019 Oct 25;16(7):1314–31. doi: 10.1080/15548627.2019.1679514 (PMC7469629; doi:10.1080/15548627.2019.1679514)

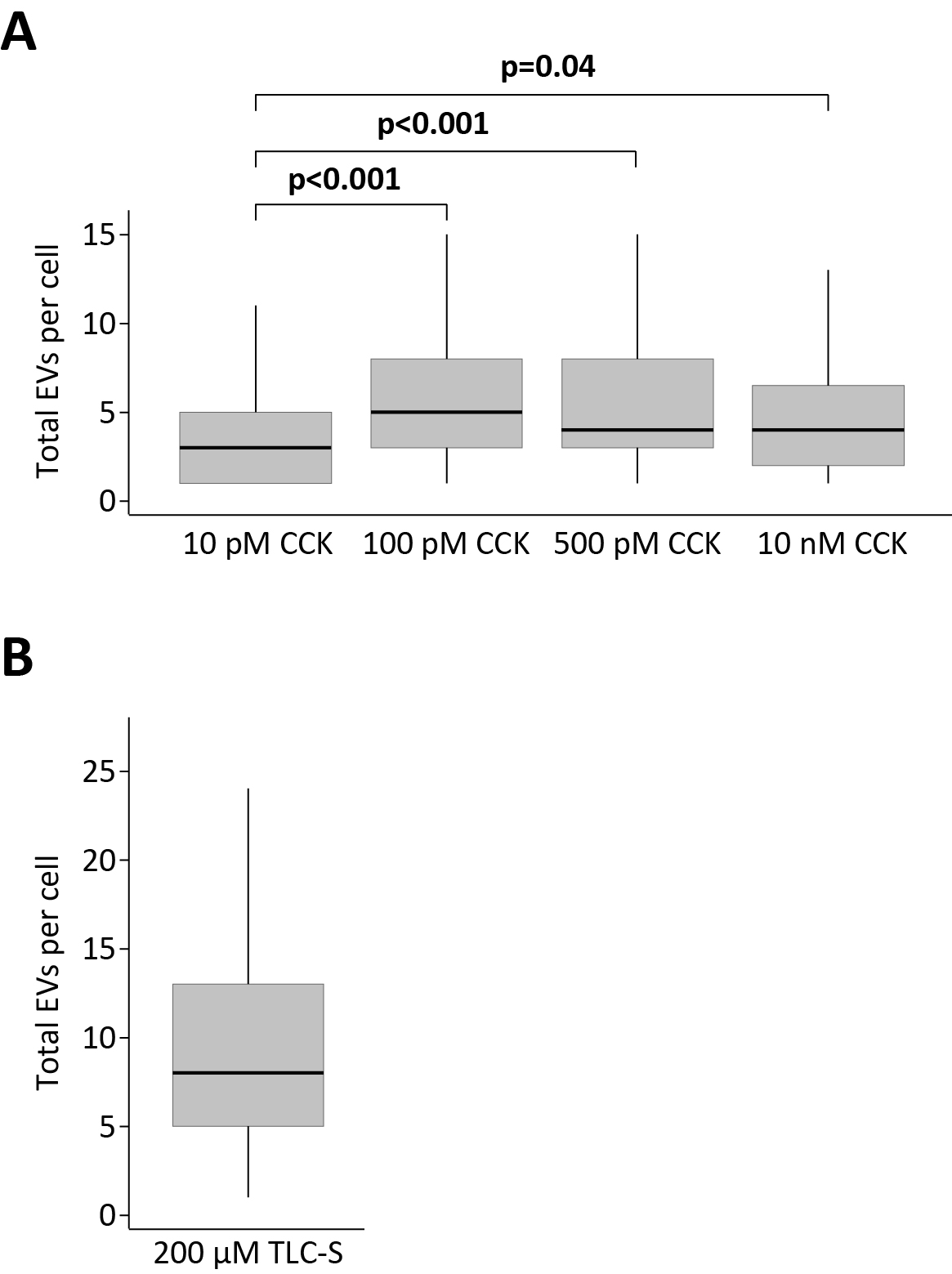

Supplement: Supplemental Material [file KAUP_A_1679514_SM7417.zip › Supplementary information/Supplementary Figure 1 for3rd Rev 12Aug VF.jpg]

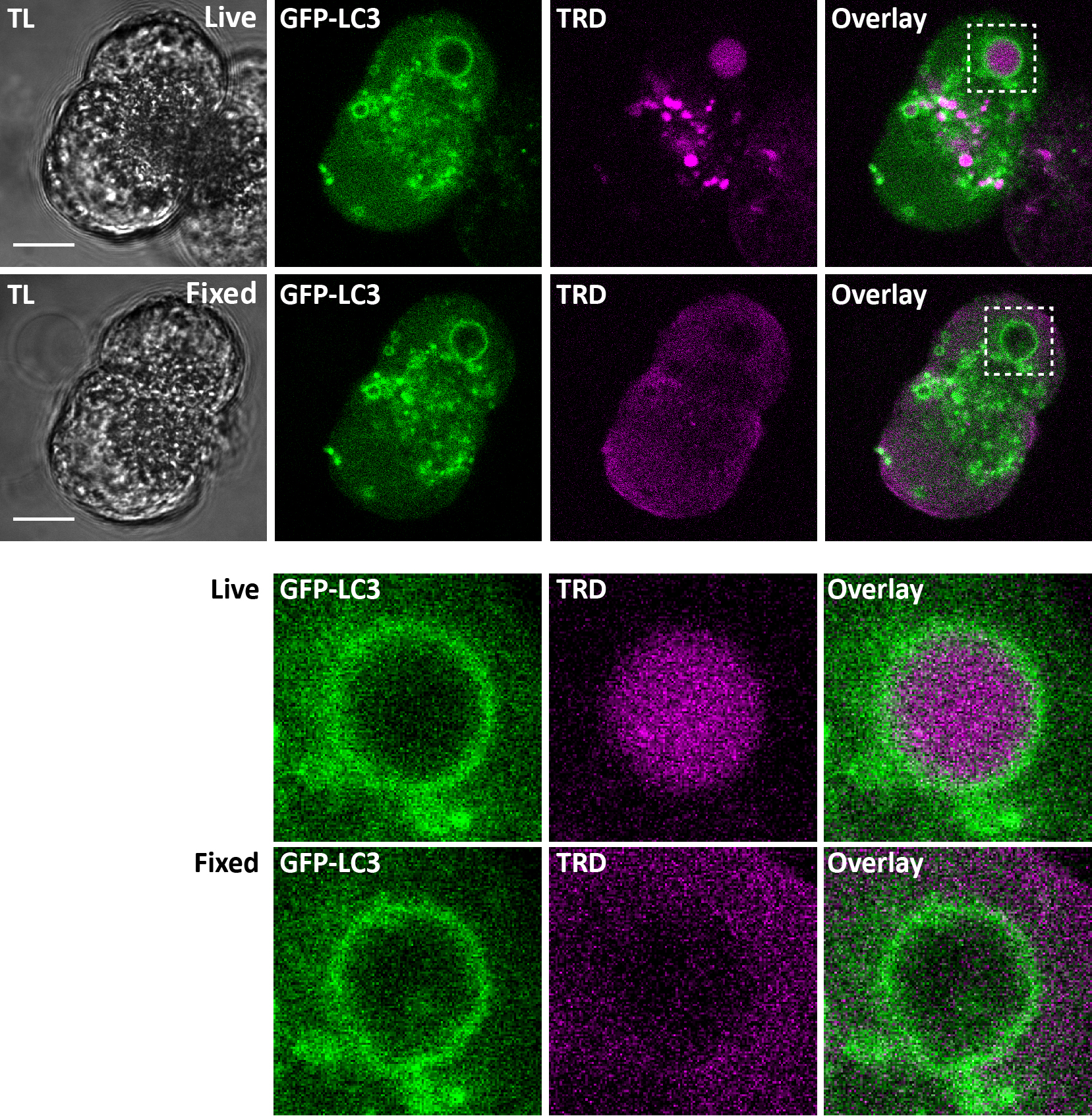

Supplement: Supplemental Material [file KAUP_A_1679514_SM7417.zip › Supplementary information/Supplementary Figure 10 .jpg]

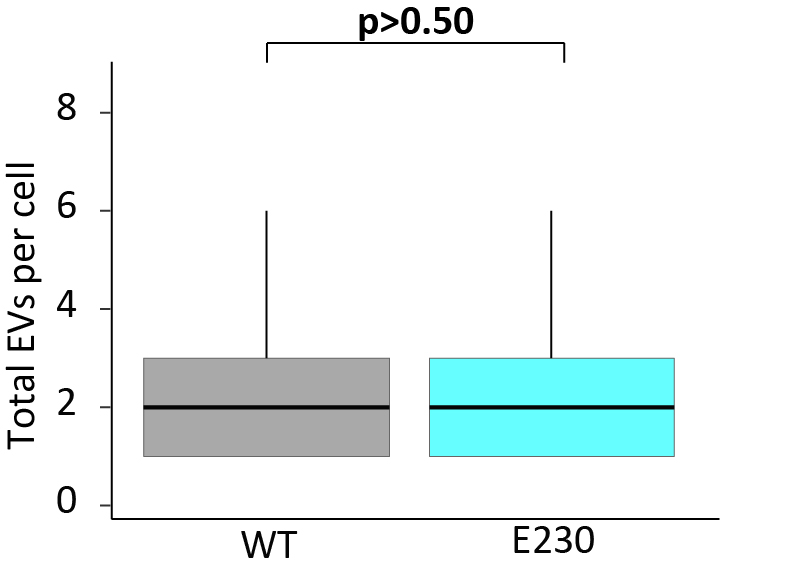

Supplement: Supplemental Material [file KAUP_A_1679514_SM7417.zip › Supplementary information/Supplementary Figure 11 .jpg]

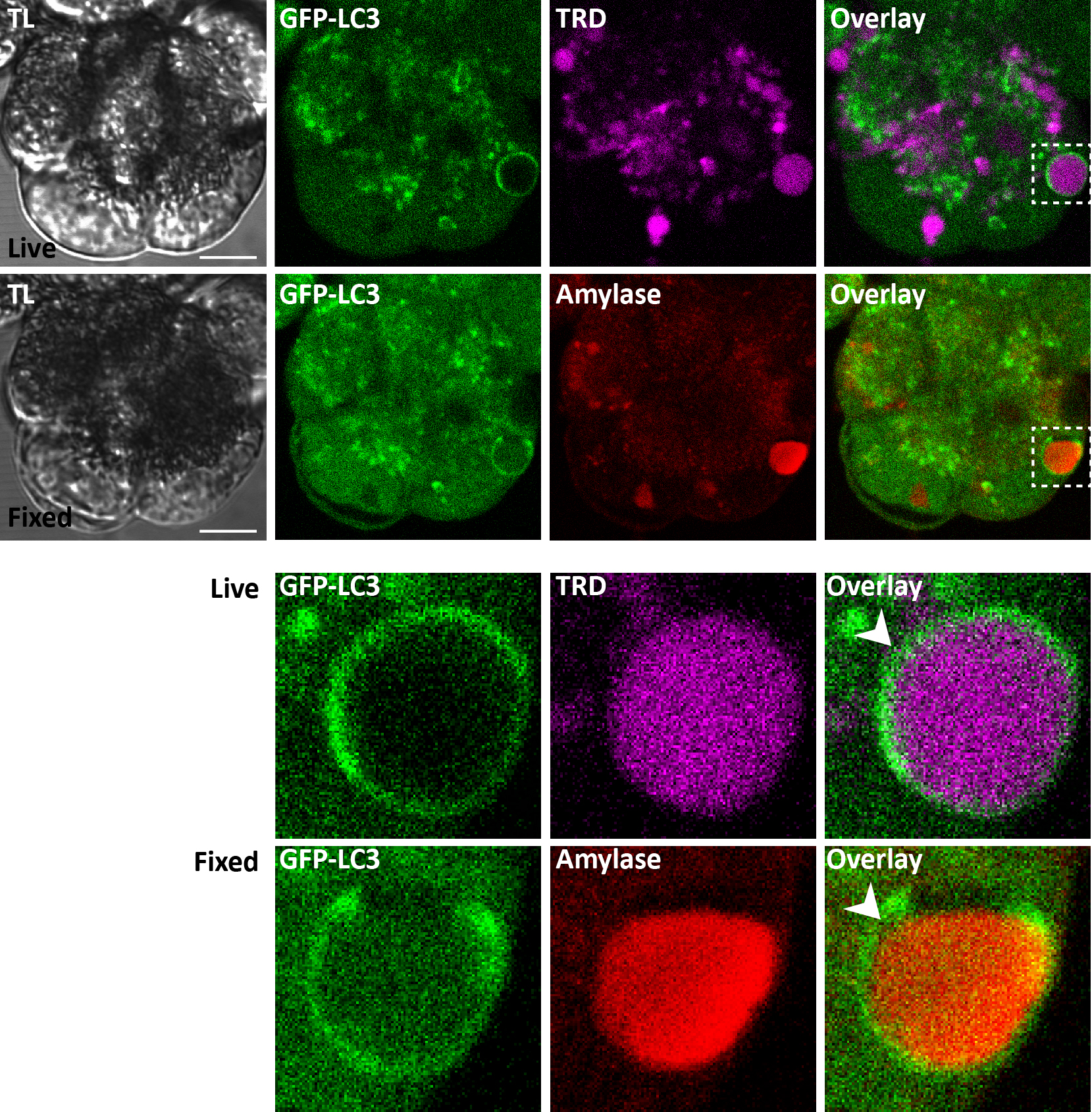

Supplement: Supplemental Material [file KAUP_A_1679514_SM7417.zip › Supplementary information/Supplementary Figure 12 .jpg]

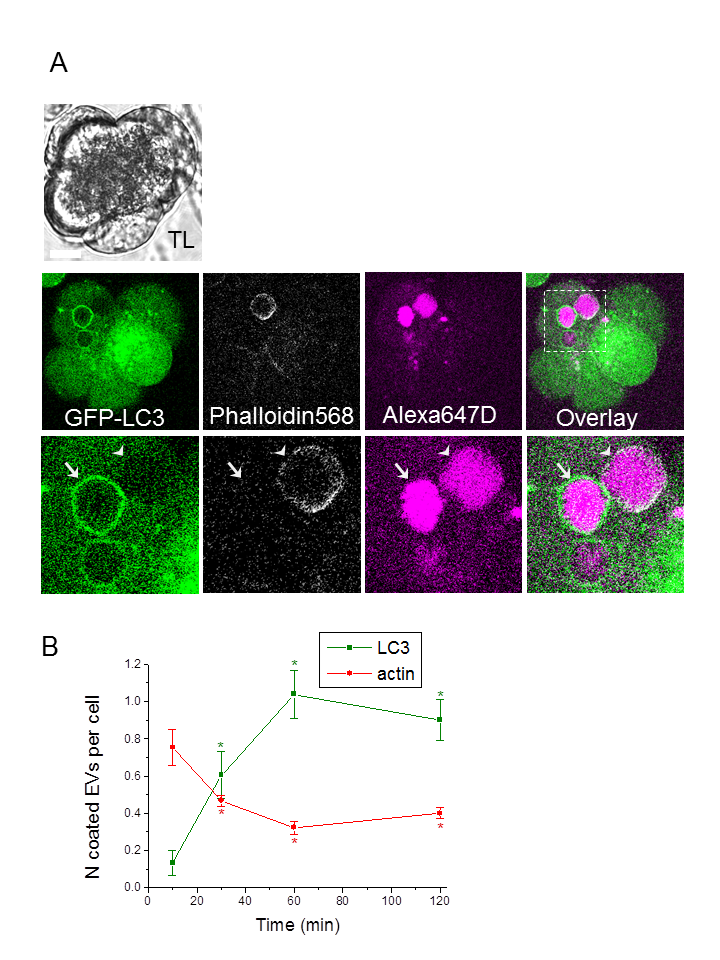

Supplement: Supplemental Material [file KAUP_A_1679514_SM7417.zip › Supplementary information/Supplementary Figure 13 .tif]

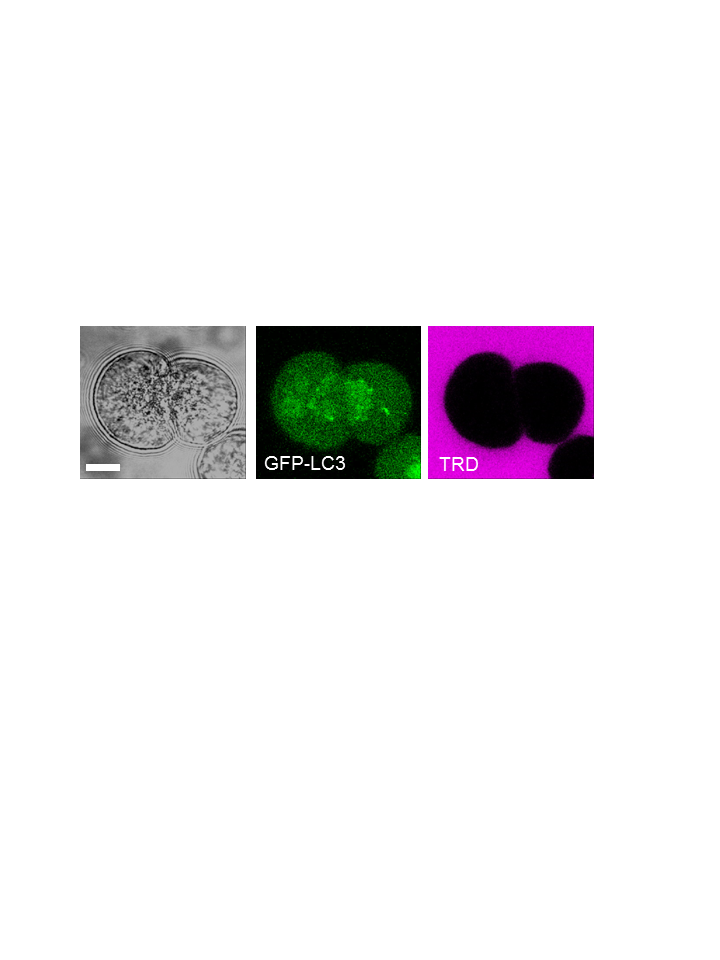

Supplement: Supplemental Material [file KAUP_A_1679514_SM7417.zip › Supplementary information/Supplementary Figure 14.tif]

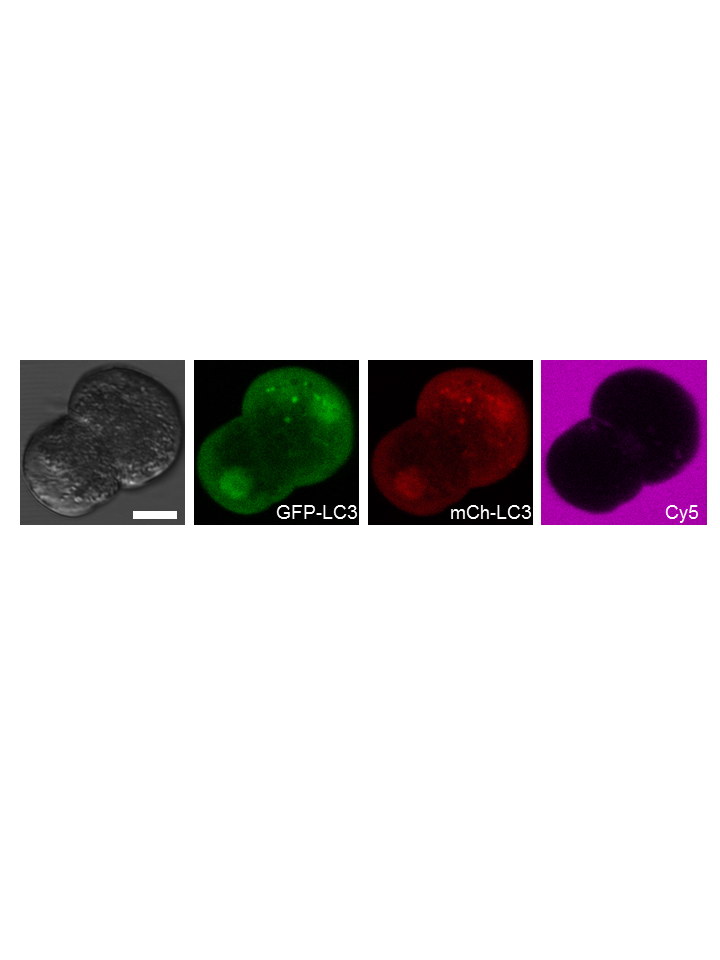

Supplement: Supplemental Material [file KAUP_A_1679514_SM7417.zip › Supplementary information/Supplementary Figure 15.tif]

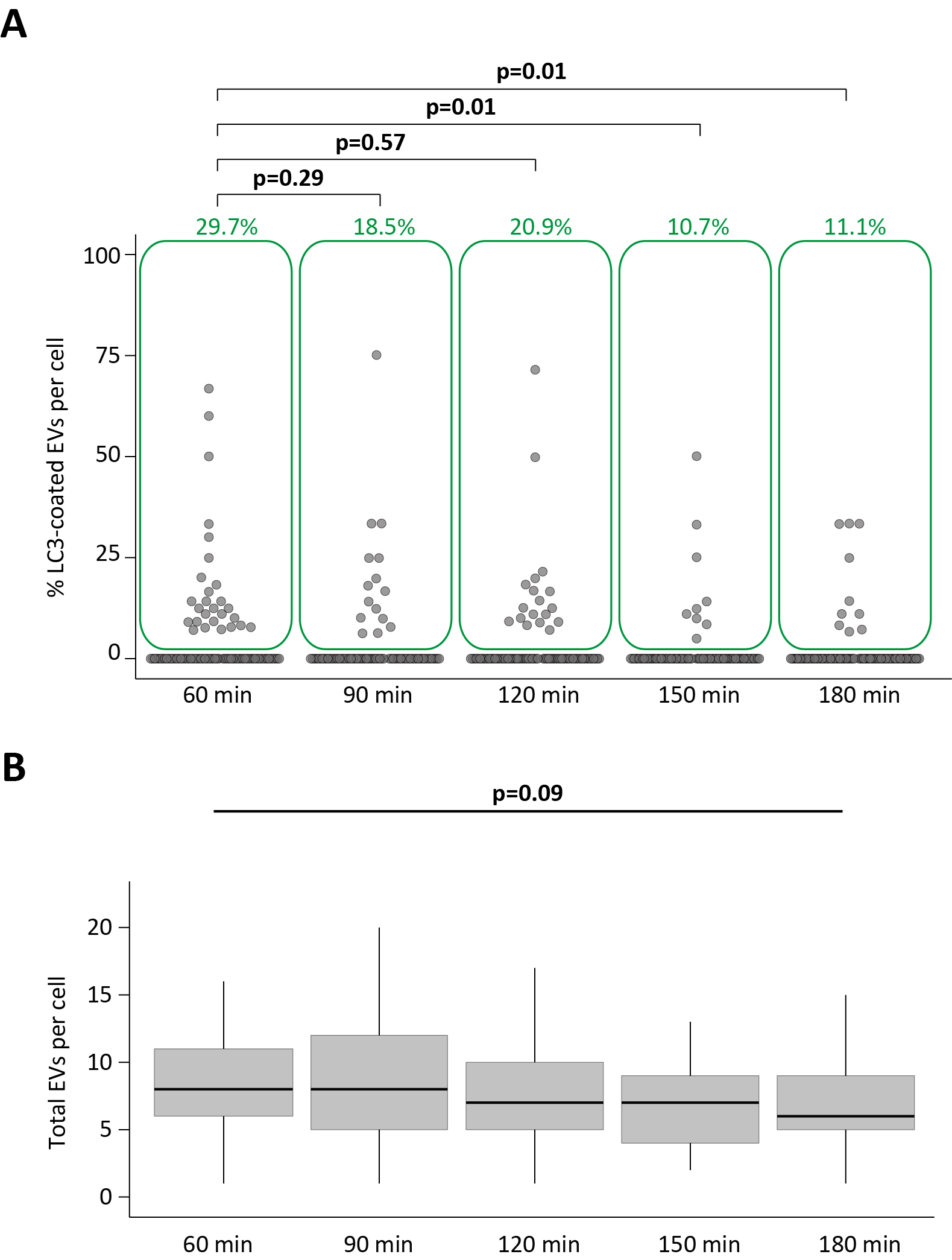

Supplement: Supplemental Material [file KAUP_A_1679514_SM7417.zip › Supplementary information/Supplementary Figure 2.jpg]

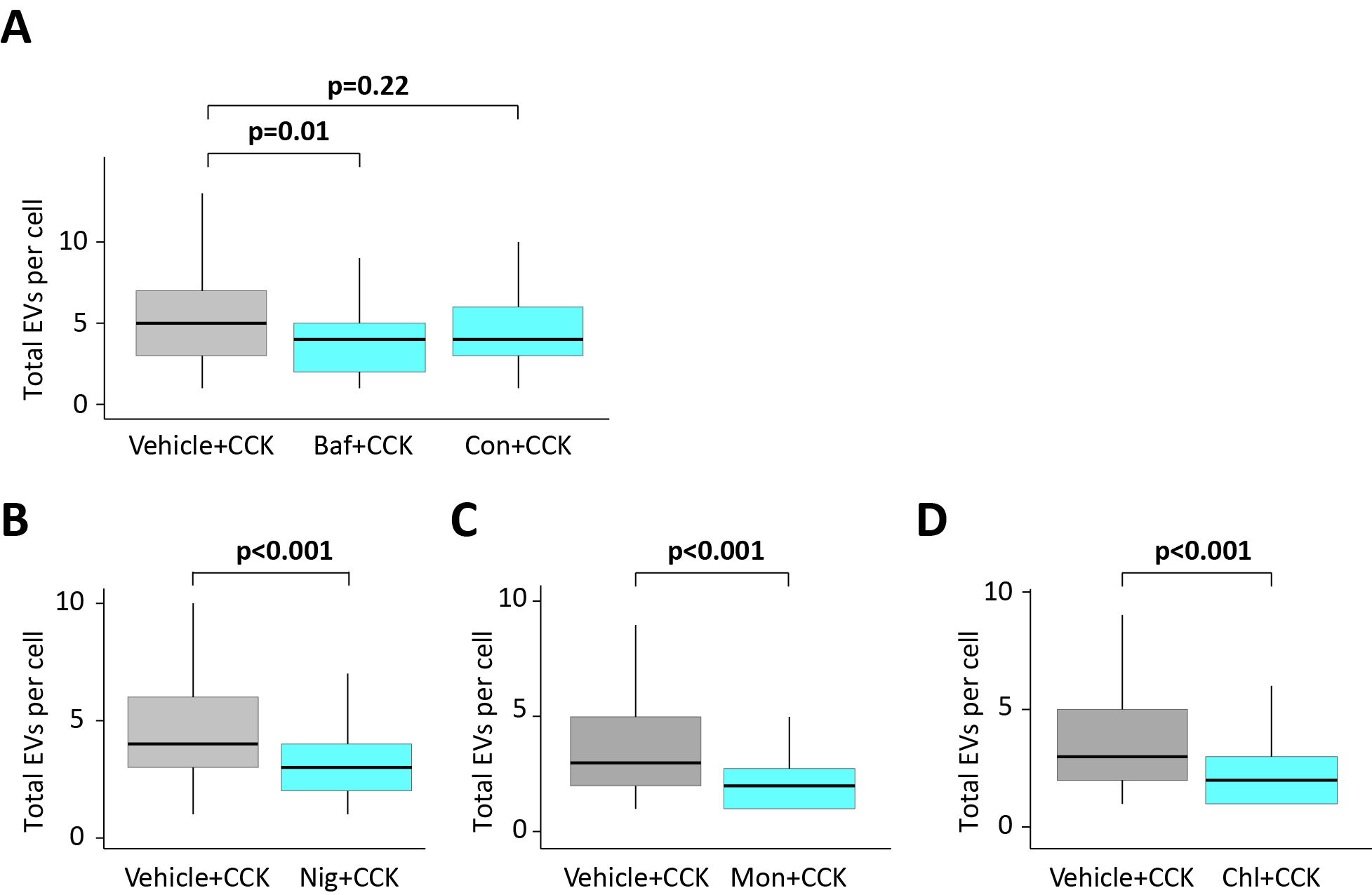

Supplement: Supplemental Material [file KAUP_A_1679514_SM7417.zip › Supplementary information/Supplementary Figure 3.jpg]

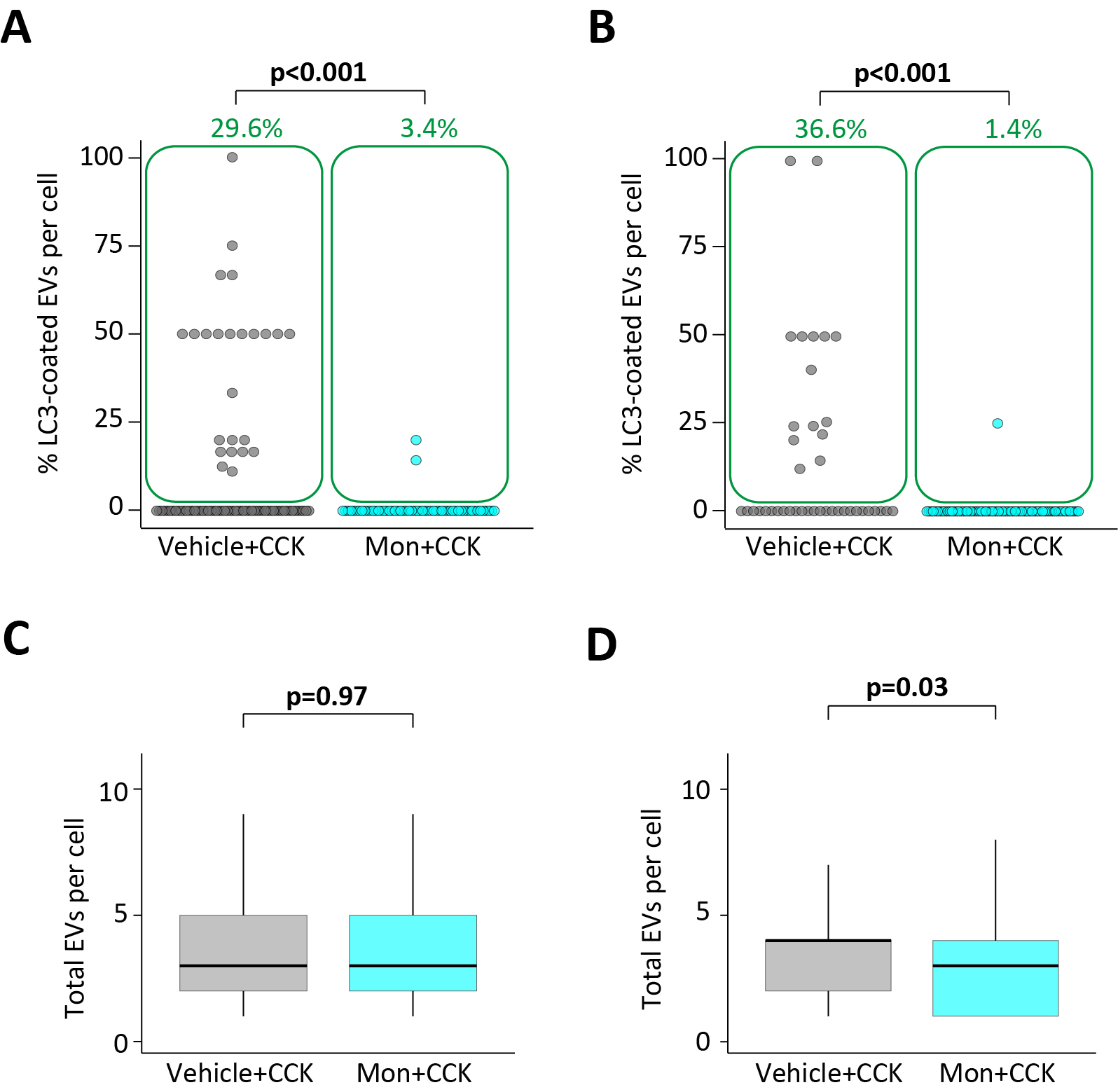

Supplement: Supplemental Material [file KAUP_A_1679514_SM7417.zip › Supplementary information/Supplementary Figure 4 .jpg]

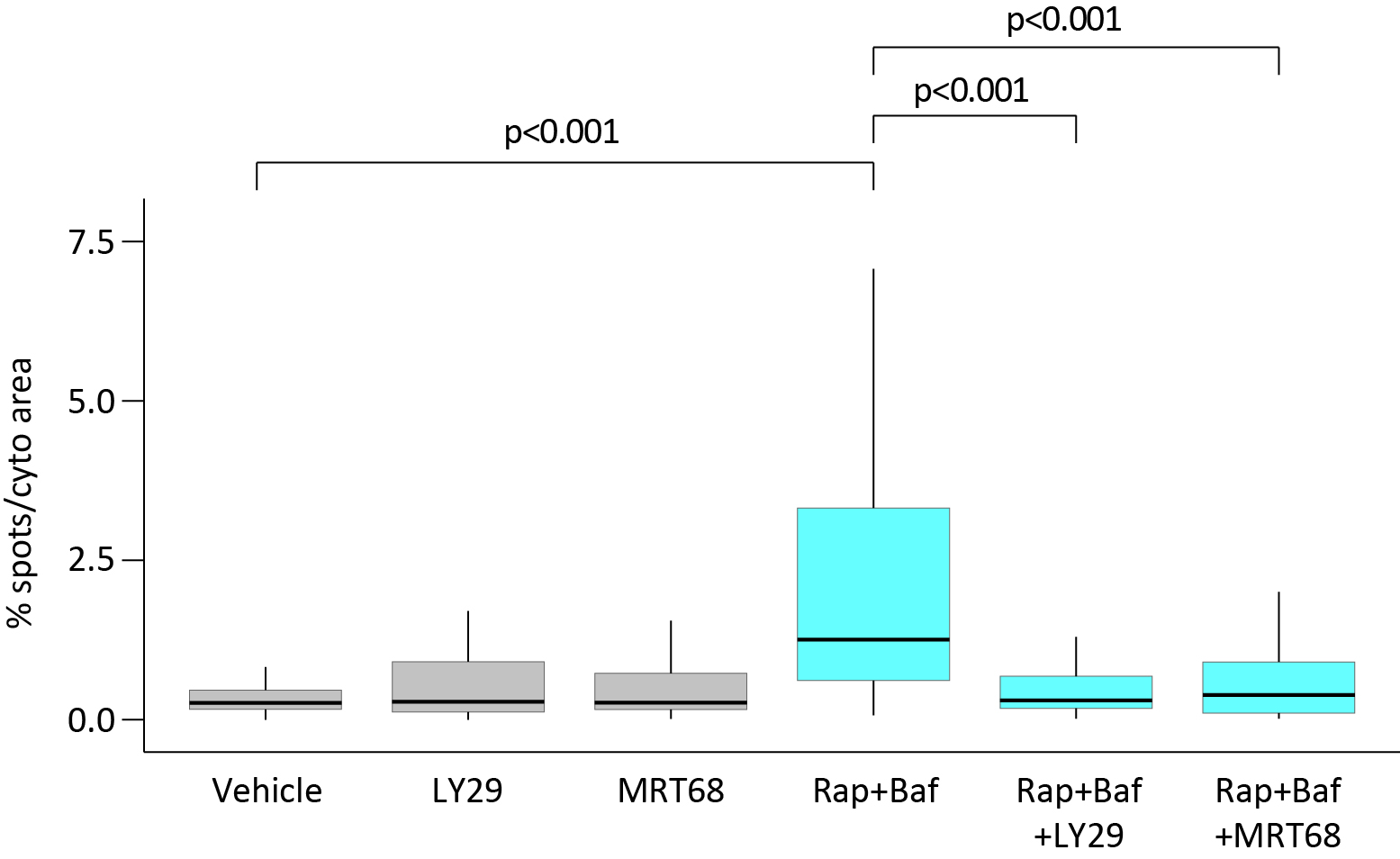

Supplement: Supplemental Material [file KAUP_A_1679514_SM7417.zip › Supplementary information/Supplementary Figure 5.jpg]

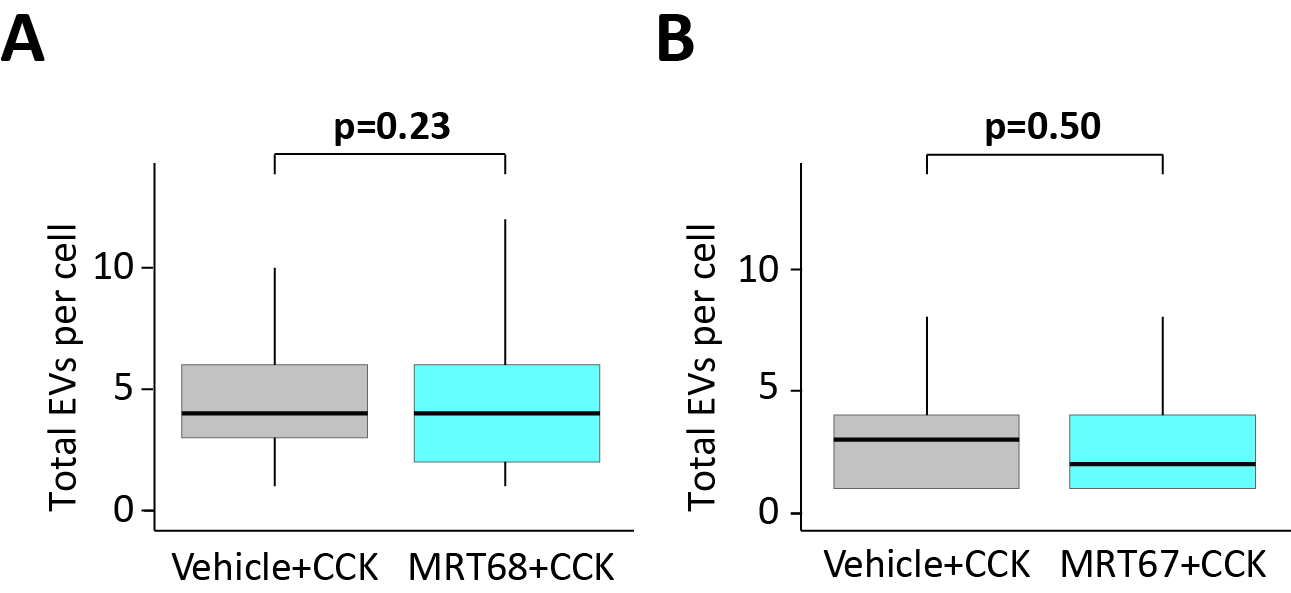

Supplement: Supplemental Material [file KAUP_A_1679514_SM7417.zip › Supplementary information/Supplementary Figure 6.jpg]

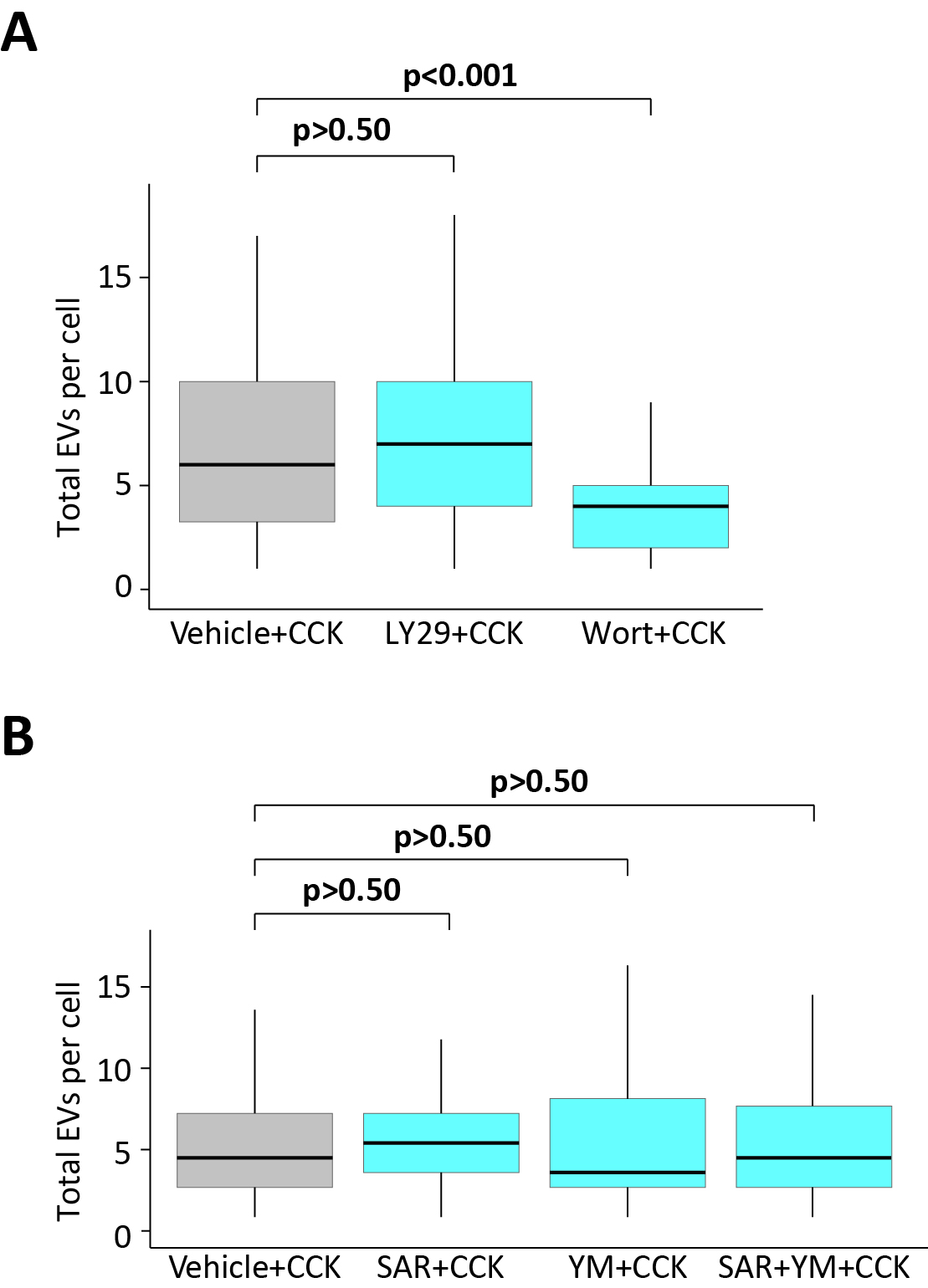

Supplement: Supplemental Material [file KAUP_A_1679514_SM7417.zip › Supplementary information/Supplementary Figure 7 .jpg]

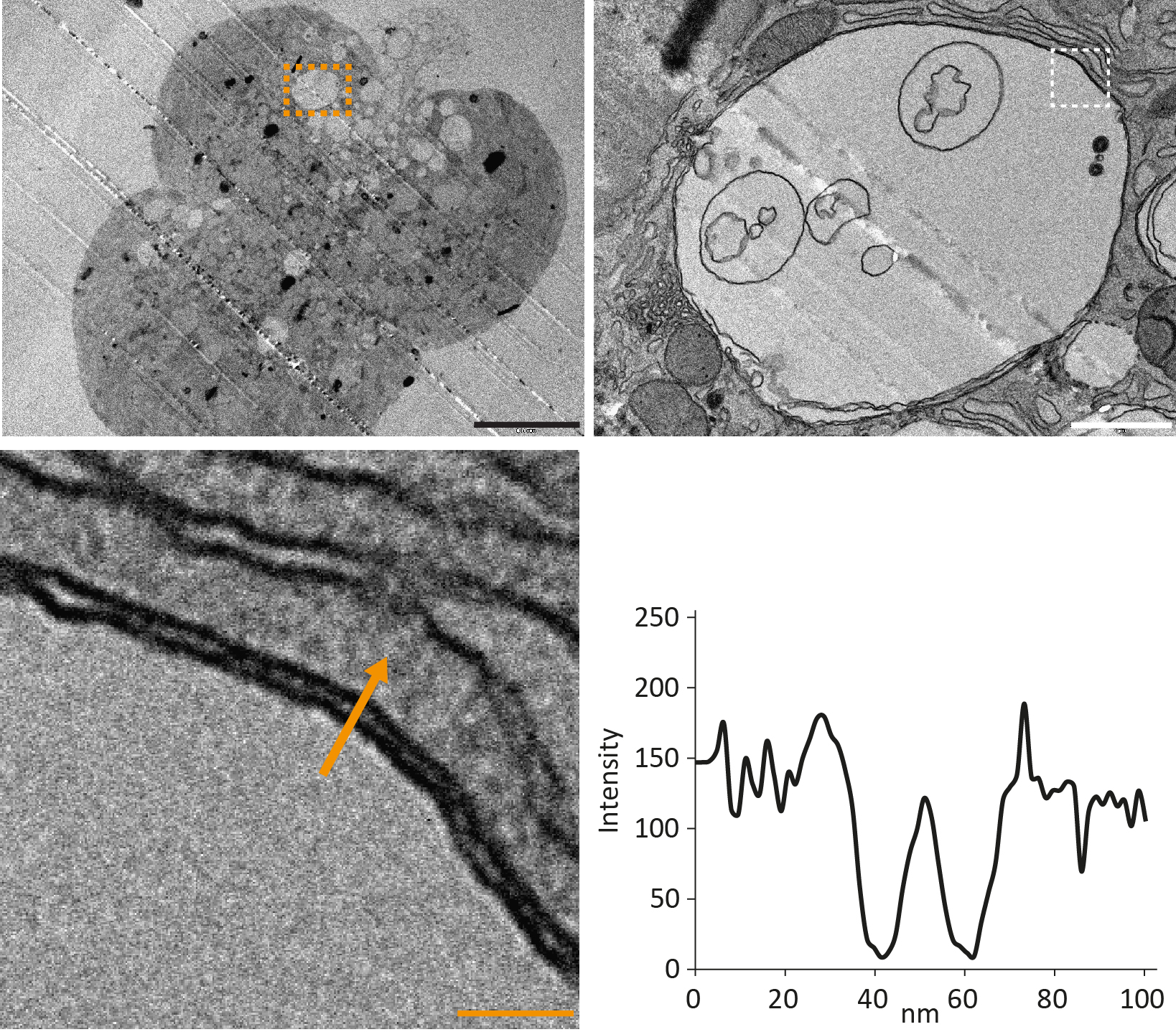

Supplement: Supplemental Material [file KAUP_A_1679514_SM7417.zip › Supplementary information/Supplementary Figure 8.jpg]

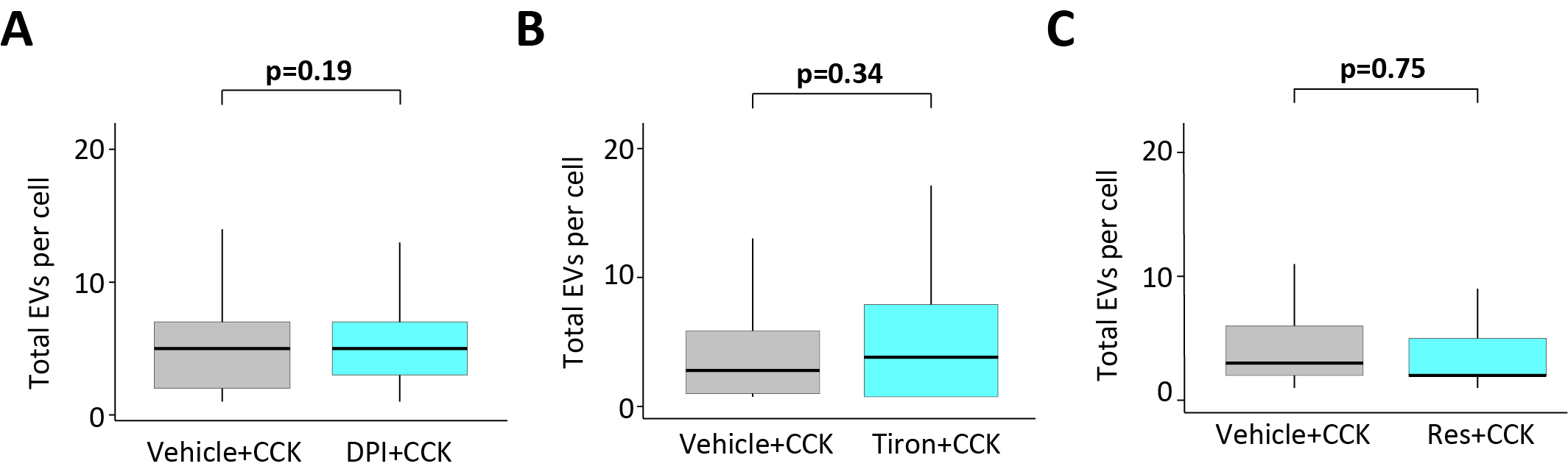

Supplement: Supplemental Material [file KAUP_A_1679514_SM7417.zip › Supplementary information/Supplementary Figure 9 .jpg]
